# Chiral Ruthenium(II) Polypyridyl Complexes: Stabilization of G-Quadruplex DNA, Inhibition of Telomerase Activity and Cellular Uptake

**DOI:** 10.1371/journal.pone.0050902

**Published:** 2012-12-07

**Authors:** Qianqian Yu, Yanan Liu, Chuan Wang, Dongdong Sun, Xingcheng Yang, Yanyu Liu, Jie Liu

**Affiliations:** 1 Department of Chemistry, Jinan University, Guangzhou, P. R. China; 2 Department of Biology, The Chinese University of Hong Kong, Hong Kong, P. R. China; Wake Forest University, United States of America

## Abstract

Two ruthenium(II) complexes, Λ-[Ru(phen)_2_(*p-*HPIP)]^2+^ and Δ-[Ru(phen)_2_(*p-*HPIP)]^2+^, were synthesized and characterized via proton nuclear magnetic resonance spectroscopy, electrospray ionization-mass spectrometry, and circular dichroism spectroscopy. This study aims to clarify the anticancer effect of metal complexes as novel and potent telomerase inhibitors and cellular nucleus target drug. First, the chiral selectivity of the compounds and their ability to stabilize quadruplex DNA were studied via absorption and emission analyses, circular dichroism spectroscopy, fluorescence-resonance energy transfer melting assay, electrophoretic mobility shift assay, and polymerase chain reaction stop assay. The two chiral compounds selectively induced and stabilized the G-quadruplex of telomeric DNA with or without metal cations. These results provide new insights into the development of chiral anticancer agents for G-quadruplex DNA targeting. Telomerase repeat amplification protocol reveals the higher inhibitory activity of Λ-[Ru(phen)_2_(*p-*HPIP)]^2+^ against telomerase, suggesting that Λ-[Ru(phen)_2_(*p-*HPIP)]^2+^ may be a potential telomerase inhibitor for cancer chemotherapy. MTT assay results show that these chiral complexes have significant antitumor activities in HepG2 cells. More interestingly, cellular uptake and laser-scanning confocal microscopic studies reveal the efficient uptake of Λ-[Ru(phen)_2_(*p-*HPIP)]^2+^ by HepG2 cells. This complex then enters the cytoplasm and tends to accumulate in the nucleus. This nuclear penetration of the ruthenium complexes and their subsequent accumulation are associated with the chirality of the isomers as well as with the subtle environment of the ruthenium complexes. Therefore, the nucleus can be the cellular target of chiral ruthenium complexes for anticancer therapy.

## Introduction

Guanine (G)-rich nucleic acid sequences tend to adopt remarkably stable secondary structures known as G-quadruplexes. [1]–[4] Human telomeres consist of simple tandem repeats of the G-tract sequence (TTAGGG/CCCTAA)*_n_*, which consists of a single-stranded tandem [TTAGGG]-repeated sequence over several hundred bases. [5], Kim et al. [6] reported that telomerase is activated in approximately 85% of cancer cells, whereas it is undetectable in most normal somatic cells. Thus, telomerase inhibition has become an attractive strategy in designing anticancer drugs [7], [8]. The folding of telomeric DNA into G-quadruplexes inhibits telomerase by locking the single-stranded RNA component template of the telomerase complex that does not recognize the quadruplex DNA [9]. Therefore, this unique telomerase activity is an ideal probe for tumor diagnosis and a target for cancer chemotherapy, with the potential for selective toxicity to cancer cells.

A number of small-molecule ligands can induce and stabilize the formation of G-quadruplex structure and inhibit telomerase activity, with some showing pronounced effects on cancer cell lines. These ligands include the natural product telomestatin, as well as cationic porphyrins, substituted acridines, polycyclic aceidines, and perylenetetrac arboxylic diimide derivatives [10]–[15]. Metal complexes, particularly those of ruthenium (Ru), have also been shown to interact selectively with G-quadruplexes and to exhibit good antitumor activities [15]–[17]. For example, the [Ru(bpy)_2_(dppz)]^2+^ complex has been identified as a distinctive “light switch.” This complex can intercalate between duplex DNA base pairs and bind to quadruplex DNA when induced by either Na^+^ or K^+^ over an i-motif, with affinities higher than those obtained for duplex binding [18]. Thomas et al. [19] reported that dinuclear tppz-based systems have high affinities for and thus are bound to quadruplex DNA at high ionic strengths through the 22-mer d(AG_3_[T_2_AG_3_]_3_)[G_3_] human telomeric sequence. However, to the best of our knowledge, only a few studies have reported on the ability of chiral enantiomers to selectively induce and stabilize G-quadruplex formation and to inhibit telomerase. One example is the enantioselective binding of the short linker-containing chiral helicene molecule to telomere repeats and its enantioselective inhibitory activity against telomerase [20]. Meanwhile, Qu et al. [21], [22] reported that the metallo supermolecular cylinders [M_2_L_3_](PF_6_)_4_ and [M_2_L_3_]Cl_4_ (M = Ni or Fe) can selectively stabilize human telomeric G-quadruplex DNA. Only the P enantiomers of these cylinders have a strong preference for G-quadruplex DNA over duplex DNA and can convert the antiparallel G-quadruplex structure to a hybrid structure in the presence of sodium.

Purified enantiomers generally exhibit very different, and even opposite, biological activities [23], [24]. Interestingly, Svensson et al. [25] reported that the Δ-enantiomer of the [Ru(phen)_2_dppz]^2+^ complex has higher DNA binding activity. Our laboratory has also previously examined the interaction of Λ-[Ru(phen)_2_(p-MOPIP)]^2+^ and Δ -[Ru(phen)_2_(p-MOPIP)]^2+^ with G-quadruplex DNA, as well as their enantioselective inhibitory effect on telomerase activity. Both complexes contain a hydrophobic methoxyl group in their aromatic heterocyclic ligands [26]. The possible correlation between the different biological activities and the isomer chiralities or the DNA complex structure remains to be determined. In addition, the biological activities of the chiral Ru complexes may be related to their ability to bind with the G-quadruplex structure. The ability of these complexes to stabilize G-quadruplex formation may also be related to their telomerase inhibition and anticancer activities. These questions motivated the investigation on the relationships between the anticancer targets of Ru complexes, DNA, and telomerase.

**Figure 1:**
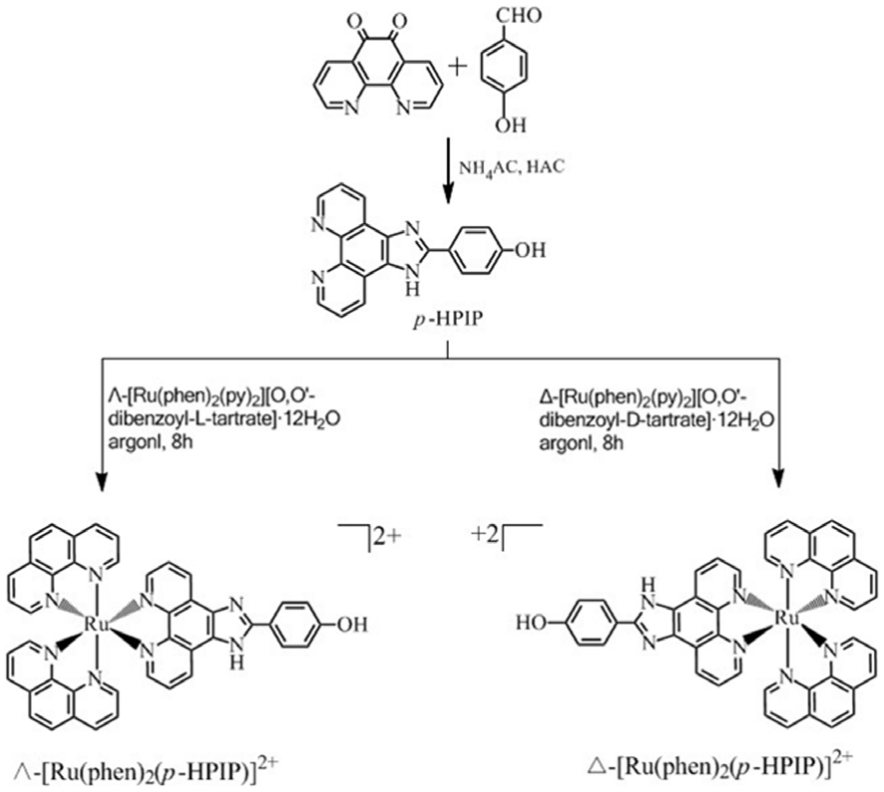
Synthesis routes for ligand and ruthenium complexes Λ-[Ru(phen)_2_(*p-*HPIP)]^2+^ and Δ-[Ru(phen)_2_(*p-*HPIP)]^2+^.

In this study, we synthesized the chiral Ru complexes Δ-[Ru(phen)_2_(*p-*HPIP)]^2+^ and Λ-[Ru(phen)_2_(*p-*HPIP)]^2+^ (*p-*HPIP = 2-(4-hydroxy-phenyl) imidazo [4,5-f] [1], [10] phenanthroline), both of which contain a hydrophilic hydroxyl group to determine systematically the effect of different aromatic heterocyclic ligands on the interaction of the complexes with G-quadruplex DNA. The synthesis route and structure of these complexes are shown in **Figure 1**.

## Experimental Sections

### 

#### Materials and chemicals

DNA oligomers 5′-G_3_(T_2_AG_3_)_3_-3′ (HTG21), the complementary cytosine rich strand: 5′-C_3_(TA_2_C_3_)_3_-3′((ssDNA), G_4_T_2_:5′-[G_4_T_2_]_3_G_4_-3′ and double-stranded competitor ds26 (5′-CAATCGGATCGAATTCGATCCGATTG-3′) were purchased from Shanghai Sangon Biological Engineering Technology & Services (Shanghai, China). Concentration of 5′- G_3_(T_2_AG_3_)_3_-3′(HTG21) and 5′- C_3_(TA_2_C_3_)_3_-3′((ssDNA) was determined by measuring the absorbance at 260 nm after melting. Single-strand extinction coefficients were calculated from mononucleotide data using a nearest-neighbour approximation [27]. The formations of intramolecular G-quadruplex was carried out as follows: the oligonucleotide samples, dissolved in different buffers, were heated to 90°C for 5 min, spontaneously cooled to room temperature, and then incubated at 4°C overnight. Buffer A:10 µM Tris-HCl, pH = 7.4; Buffer B:10 µM Tris-HCl, 100 µM NaCl, pH = 7.4; Buffer C:10 µM Tris-HCl, 100 µM KCl, pH = 7.4. Stock solutions were stored at 4°C and used after no more than 4 days. Further dilution was made in the corresponding buffer to the required concentrations for all the experiments. All reagents and solvents were purchased commercially and used without further purification unless specially noted and Ultrapure MilliQ water (18.2 mX) was used in all experiments.

#### Physical measurements

Elemental analyses (C, H and N) were carried out with a Perkin–Elmer 240C elemental analyzer. ^1^H NMR spectra were recorded on a Varian Mercury-plus 300 NMR spectrometer with DMSO-d6 as a solvent and SiMe_4_ as an internal standard at 300 MHz at room temperature. An LCQ electrospray mass spectrometer (ESMS, Finnigan) was employed for the investigation of charged metal complex species in CH_3_CN solvent. Emission spectra were measured on a recorded on Perkin-Elmer Lambda-850 spectrophotometer with excitation at 460 nm, and circular dichroism (CD) spectra were measured on a Jasco J-810 spectropolarimeter.

#### Synthesis and characteristics of ligands and complexes

Ru^II^ chloride hydrate (Alfa Aesar), 1,10-phenanthroline -5,6-dione and *p-*HPIP (2-(4-hydroxy-phenyl) imidazo[4,5-f] [1], [10] phenanthroline) were obtained from Sigma. cis-[Ru(phen)_2_Cl_2_]·2H_2_O, cis-[Ru(phen)_2_(py)_2_]Cl_2_, Δ-[Ru(phen)_2_(py)_2_] [*O,O′*-dibenzoyl -D-tartrate]·12H_2_O and Λ-[Ru(phen)_2_(py)_2_] [*O,O′*-dibenzoyl -L-tartrate]·12H_2_O were prepared and characterized according to the literature [28]. (*p-*HPIP) was also prepared according to the literature [29].

#### Synthesis of Λ-[Ru(phen)_2_(*p-*HPIP)](ClO_4_)_2_⋅2H_2_O (Λ-Ru)

This complex was synthesized in a manner identical to that described for Δ-[Ru(phen)_2_(*p-*HPIP)] (ClO_4_)_2_⋅2H_2_O With Λ-[Ru(phen)_2_(py)_2_] [*O,O′*-dibenzoyl –L- tartrate]·12H_2_O and *p-*HPIP, and exhibited an identical 1HNMR spectrum. Yield: 150 mg, 75.00%. ^1^H NMR(DMSO-d_6_, dppm): 8.00(1H, d); 8.05(1H, d); 7.67(1H, 2d); 7.72(1H, 2d); 8.67(1H, d); 8.70(1H, d); 8.29(2H, s); 7.88(1H, d); 7.70(1H, 2d);9.11(1H, d); 8.13(1H, d); 6.81(1H, d); 3.14(Me, s) (**Figure S4**). ES-MS of the ClO_4_ salt in MeCN: m/z 773.3(M-2ClO_4_-H); 387.3 ((M-2ClO_4_)/2). UV-Vis (λ (nm), ε (10^4^ M^−1^ cm^−1^)) (CH_3_OH): 263 (0.53), 346 (0.36), 6463 (0.11)(**Figure S5**). CD (λ (nm), Δε (M^−1^ cm^−1^)) (CH_3_OH)): 470 (5.03), 307(1.14), 269 (4.15) (**Figure S6**).

#### Synthesis of Δ-[Ru(phen)_2_(*p-*HPIP)](ClO_4_)_2_⋅2H_2_O (Δ-Ru)

Δ-[Ru(phen)_2_(py)_2_][*O, O′*-dibenzoyl-D-tartrate]·12H_2_O (0.22g, 0.2 mmol), *p-*HPIP (0.12 g, 0.36 mmol) were added to 20 ml ethylene glycol–water(9∶1, v/v). The mixture was refluxed for 6 h under an argon atmosphere. The cooled reaction mixture was diluted with water (40 ml) and filtered to remove solid impurities. Ammonium hexafluorophosphate was added to the filtrate. The precipitated complex was dried, dissolved in a small amount of acetonitrile, and purified by chromatography over alumina, using MeCN–toluene (2∶1, v/v) as eluent, yield: 140 mg, 70.01%. ^1^H NMR (DMSO-d_6_, d ppm): 9.02(2H, d); 8.78(4H, d); 8.39(4H, s); 8.13(2H, d); 8.12(2H, d); 8.10(2H, d); 8.08(2H, d); 7.78(6H, m); 6.95(2H, d). ES-MS of the ClO_4_ salt in MeCN: m/z 773.6(M-2ClO_4_-H); 387.3 ((M-2ClO_4_)/2). UV-Vis (λ (nm), ε (10^4^ M^−1^ cm^−1^)) (CH_3_OH): 263 (0.80), 282 (0.54), 464 (0.16) (**Figure S7**). CD (λ (nm), Δε (M^−1^ cm^−1^)) (CH_3_RU)): 470 (−0.49), 307(−1.19.), 269 (−4.30) (**Figure S6**).

### Synthesis of [Ru(phen)_2_(*p-*HPIP)] (ClO_4_)_2_⋅2H_2_O (Λ/Δ-Ru)

[Ru(phen)_2_Cl_2_] ⋅2H_2_O (0.12 g, 0.2 mmol) and *p-*HPIP(0.12 g, 0.36 mmol) were added to 20 ml ethylene glycol–water(9∶1, v/v). The mixture was refluxed for 6 h under an argon atmosphere. The cooled reaction mixture was diluted with water (40 ml) and filtered to remove solid impurities. Ammonium hexafluorophosphate was added to the filtrate. The precipitated complex was dried, dissolved in a small amount of acetonitrile, and purified by chromatography over alumina, using MeCN–toluene (2∶1, v/v) as eluent, yeild: ^1^H NMR(DMSO-d_6_, d ppm): 8.00(1H, d); 8.05(1H, d); 7.67(1H, 2d); 7.72(1H, 2d); 8.67(1H, d); 8.70(1H, d); 8.29(2H, s); 7.88(1H, d); 7.70(1H, 2d);9.11(1H, d); 8.13(1H, d); 6.81(1H, d); 3.14(Me, s) (**Figure S7**). ES-MS of the ClO_4_ salt in MeCN: m/z 773.3(M-2ClO_4_-H); 387.3 ((M-2ClO_4_)/2).

#### Absorption spectra studies

Electronic spectra were recorded on a Shimadzu UVPC-3000 spectrophotometer. Spectroscopic titrations were carried out at room temperature to determine the binding capability affinity between DNA and each enantiomer. Initially, 3000 µL solutions of the blank buffer and the ruthenium complex sample (2 µM) were placed in the reference and sample cuvettes (1 cm path length), respectively, and then the first spectrum was recorded in the range 200–600 nm. During the titration, aliquots (1–10 µL) of buffered DNA solution (concentration of 5–10 µM in base pairs) was added to each cuvette to eliminate the absorbance of DNA itself, and the solutions were mixed by repeated inversion. After mixing for 5 min, the absorption spectra were recorded. The titration processes were repeated until there was no change in the spectra for at least four titrations indicating binding saturation had been achieved. The changes in the metal complex concentration due to dilution at the end of each titration were negligible. The UV-Vis is titrations for each sample were repeated at least three times.

#### Emission measurements

Emission measurements were carried out on a JASCOFP-6500 spectrofluorometer at 20°C. For luminescence titrations a 3000 µL aliquot of the sample solution in a 1 cm path length quartz cuvette was loaded into the fluori-meter sample block, After 5 min to allow the cell to equilibrate, the first spectrum was recorded, and then 1–10 µL of DNA solution (5–10 µM in base pairs) was added to the sample cell, followed by thorough mixing. After 5 min, the spectrum was taken again. Lifetime spectrometer at room temperature with excitation wavelength 460 nm, Exslit 5.00 nm, and emslit 1.50 nm. The titration processes were repeated until there was no change in the spectra for at least four titrations indicating binding saturation had been achieved. The luminescence titrations for each sample were repeated at least three times.

#### Circular dichroism measurements

All CD experiments were performed at an ambient temperature in aerated buffer solutions in 10 mM Tris-HCl buffer, 100 mM NaCl at pH = 7.4. CD titrations were carried out as follows: concentrated DNA (5–10 µM in base pairs) was added in aliquots to solutions containing Ru(II) complex. All solutions were mixed thoroughly and allowed to equilibrate for 6 min before data collection. The titration process was repeated several times until no change was observed. It showed that binding saturation was achieved. The CD spectra were recorded on a Chirascan (Applied Photophysics) spectrophotometer, using 0.5/1.0 s-per-points from 220 to 350 nm and 1 nm bandwidth at a temperature of 25°C. The CD spectra were obtained by averaging three scans. The instrument was flushed continuously with pure evaporated nitrogen throughout the experiment.

#### Gel Mobility Shift Assay

The Oligonucleotide at 10 µM was heated to 95°C for 10 min in 10 mM Tris/1 mM EDTA buffer containing 100 mM KCl (pH 7.4). After the DNA was cooled to room temperature, a 2 µL stock solution of the metal complex was added and each sample to produce the specified concentrations. The reaction mixture was incubated for 4 h at room temperature, then loaded onto a native 12% acrylamide vertical gel (1/19 bisacrylamide) in Tris borate EDTA (TBE) buffer, supplemented with 20 mM KCl. After these, each mixture added 8 µL of loading buffer (30% glycerol, 0.1% bromophenol blue, and 0.1% xylene cyanol). Ten microliter solution of each sample were subsequently analyzed by native 12% PAGE (the gel was pre-run for 30 min). Electrophoresis proceeded for 15 h in TBE running buffer containing 20 mM KCl at 4°C. The gels were silver-stained to visualized.

#### FRET assay

The two double-dye labelled oligonucleotide F21T(5′-FAM-G_3_[T_2_AG_3_]_3_-TAMRA-3′) was diluted in Tris-HCl buffer (10 mM, pH 7.4) containing 60 mM KCl and then annealed by heating to 92°C for 5 min, followed by cooling slowly to room temperature overnight. Emission readings were taken at an interval of 1°C over the range 30–95°C, with a constant temperature being maintained for 30 s prior to each reading to ensure a stable value. To test the binding selectivity of the compound to the quadruplex structure, we added various concentrations of competitors: double-stranded DNA (self-complementary ds26 DNA: 5′-GTTAGCCTAGCTTAAGCTA GGCTAAC-3′). Final analysis of the data was carried out using Origin 7.0(Origin Lab Corp.).

#### Cell culture

Cells were cultured in RPMI 1640 medium supplemented with 10% heat inactivated fetal bovine serum, 100 µg/ml penicillin, and 100 µg/ml streptomycin. Cells were maintained at 37°C in a 5% CO_2_ incubator, and the media were changed twice weekly.

#### MTT assay

The cytotoxicity of the complexes was evaluated by cell viability and determined by measuring the ability of cells to transform MTT to a purple formazan dye [30]. Cells were incubated at 37°C under a 5% CO_2_ atmosphere, and seeded in a 96-well plates (1.0×10^3^/well) in growth medium (100 µL) and incubated at 37°C in 5% CO_2_ atmosphere for 24 h. Then the cells were treated with various concentrations of complexes in a mixture of growth medium/DMSO (99∶1, v/v); The cells was incubated at 37°C under a 5% CO_2_ atmosphere for 48 h, MTT (100 µl of 5 mg/ml) was added to each well, and then the plates were further incubated for 4 h, each cell was added in 100 µl cell lysate. After 12 h at 37°C, The absorbance of the solutions at 580 nm was measured with a microplate-reader (the absorbance of the complexes at this wavelength can be neglected [31], [32]). The IC_50_ values of the complexes were determined by plotting the percentage viability versus concentration on a logarithmic graph and reading off the concentration at which 50% of cells viable relative to the control.

#### PCR stop assay

Sequences of the tested oligomers were HTG21 (5′-G3(T2AG3)3-3′) and the corresponding complementary sequence (HTG21rev, ATCGCT_2_CTCGTC_3_TA_2_C_2_). The reactions were performed in 1×PCR buffer, containing 10 µM of each oligonucleotide, 0.16 µM dNTP, 2.5 U Taq polymerase, and different concentrations of complexes. Reaction mixtures were incubated in a thermocycler with the following cycling conditions: 94°C for 3 min, followed by 30 cycles of 94°C for 30 s, 58°C for 30 s, and 72°C for 30 s. PCR products were then analysed on 15% nondenaturing polyacrylamide gels in 1× TBE and silver stained.

#### TRAP Assaay

Telomerase extract was prepared from Hela cells. TRAP assay was performed by using a modification of the TRAP assay [33], [34]. Every reaction was performed in a final 50 µL reaction volume composed of a 45 µL reaction mix containing 20 µM Tris–HCl (pH 8.0), 50 µM deoxynucleotide triphosphates, 1.5 µM MgCl_2_, 63 µM KCl, 1 µM EGTA, 0.005%, Tween 20, 20 µg/mL BSA, 3.5 pmol of primer HTG21 (5′-G_3_(T_2_AG_3_)_3_-3′), 18 pmol of primer TS(5′-A_2_TC_2_GTCGAGCAGAGT_2_-3′), 22.5pmol of primer CXext(5′-GTGC_3_T_2_AC_3_T_2_AC_3_T_2_AC_3_TA_2_-3′), 7.5 pmolof primer NT(5′-ATCGCTCTCG_2_C_2_TTT_4_-3′), 0.01 amol of TSNT internal control (5′-A_2_TC_2_GTCGAGCAGAGT_2_AA_4_AG_2_C_2_GAGA_2_GCGAT-3′), 2.5 U of Taq DNA polymerase, and 100 ng of telomerase. 5 µL of compounds or distilled water was added under a volume PCR amplification was performed 30 cycles at 92°Cfor 30 s, 52°C for 30 s, and 72°C for 30 s and incubated for 30 min at 30°C. After amplification, 8 µL of loading buffer (containing 5×Tris-Borate-EDTA buffer (TBE buffer), 0.2% bromophenol blue, and 0.2% xylene cyanol) was added to the reaction. A 15 µL aliquot was loaded onto a 16% non-denaturing acrylamide gel (19∶1) in 1×TBE buffer and electrophoresed at 200 V for 1 h. Gels were fixed and then stained with AgNO_3_.

#### Cellular Uptake

HepG2 cells in growth medium were seeded in 35 mm tissue culture dishes and incubated at 37°C under a 5% CO_2_ atmosphere until 70%confluence. The culture medium was removed and replaced with medium (final DMSO concentration, 1% v/v) containing the Ru(II) complexes at 20 µM. After incubation for 12, 24, 36 h respectivly, the cell layer was trypsinized and washed twice with cold PBS. The samples were raised in 500 µL of cold PBS and analyzed by a FACSCalibur flow cytometer immediately. The samples were collected in FL2 channel (excitation at 488 nm, and the number of cells analyzed for each sample was 10000**)** [35].

#### Laser Confocal Microscopy Image Analysis

For achieving laser confocal images, HepG2 cells were grown on a laser confocal microscopy 35 mm^2^ culture dish at a density of 1.0×10^4^ cells and maintained culture with at 37°C under a 5% CO_2_ atmosphere for 24 h, then was added in the cell layer. Cells were transfected with the complexes Λ-[Ru(phen)_2_(*p-*HPIP)]^2+^ and Δ-[Ru(phen)_2_(*p-*HPIP)]^2+^ at a concentration of 20 µM and incubated for different time intervals (24 h and 48 h). After the transfection, the media were removed and the cell layer was washed 3 times with 1×PBS. Then, the cell layer was trypsinized and added up to 3 mL PBS. Confocal images were analyzed by a Leica TCS SP5 confocal microscope (Leica Microsystems, Wetzlar, Germany) using a planapochromate 63×/NA 1.4 oil immersion objective. The confocal microscope was equipped with an ArKr laser which was used to excite Ru^II^ (488 nm excitation, detection at 560–615 nm (green) and 625–754 nm (red)). Meanwhile, the cell nuclei were stained with Hoechst 33342 solution for 10 min (5 mg mL^−1^).

## Results and Discussion

### 

#### Fluorescence selectivities of Ru complexes to G-quadruplex structures

**Figure 2:**
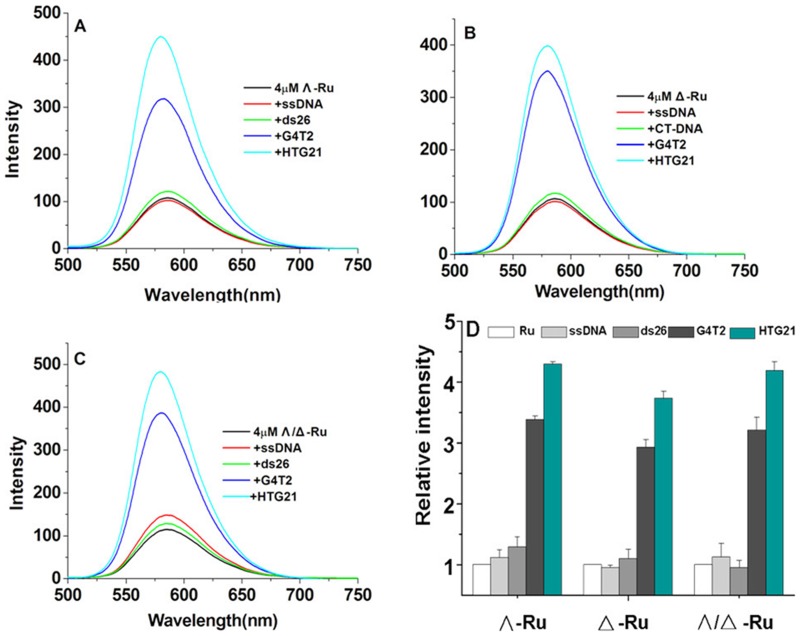
Selectivity of the Ru complex between quadruplex DNA and non-quadruplex DNA. The concentration of the ruthenium complex was 4 µM, and the concentration of the DNA was 8 µM in Tris-HCl (pH = 7.4) and KCl (100 mM): a) Λ-[Ru(phen)_2_(*p-*HPIP)]^2+^, b) Δ-[Ru(phen)_2_(*p-*HPIP)]^2+^, c) Λ/Δ -[Ru(phen)_2_(*p-*HPIP)]^2+^. d)Relative emission strength of Λ-[Ru(phen)_2_(*p-*HPIP)]^2+^, Δ-[Ru(phen)_2_(*p-*HPIP)]^2+^, and Λ/Δ -[Ru(phen)_2_(*p-*HPIP)]^2+^.

The binding affinities of these chiral Ru complexes for different DNA structures were investigated via emission spectroscopy. Two different G-quadruplex sequences, HTG21 and G_4_T_2_, were selected for this study [36]. Meanwhile, a complementary oligonucleotide of telomeric DNA (ssDNA) and double-stranded DNA (ds26) were selected as the other DNA structures. All measurements were performed in a Tris buffer containing 10 mM Tris-HCl and 100 mM KCl. The different emission spectra are illustrated in **Figure 2**. Only a slight increase in emission was observed in the presence of ds26, whereas a decrease in fluorescence was observed in the presence of ssDNA These results are attributed to the inability of ssDNA and ds26 to fold into a quadruplex even in the presence of monovalent cations. However, the emission significantly increased in the presence of the DNA quadruplexes HTG21 and G_4_T_2_. The emission response of Λ-[Ru(phen)_2_(*p-*HPIP)]^2+^ with G-quadruplexes was approximately four times higher than that with ds26. This can be very obviously enucleated that these chiral complexes exhibited high selectivity for quadruplexes over duplexes, particularly for the human telomeric DNA HTG21.We further examined the interaction between the chiral complexes and HTG21.

#### Absorption and emission luminescence spectroscopic studies

Electronic absorption spectroscopy is one of the most useful techniques in DNA-binding studies. Hypochromism and bathochroism are usually observed when a complex binds to DNA through intercalation because of the strong stacking interaction between an aromatic chromophore and the DNA base pairs in the intercalation mode. In general, the extent of hypochromism indicates the intercalative binding strength [37].

The absorption spectra of the chiral Ru(II) complexes Λ-[Ru(phen)_2_(*p-*HPIP)]^2+^ and Δ-[Ru(phen)_2_(*p-*HPIP)]^2+^ are shown in **Figure S1**. Hypochromism increased was accompanied by a red shift in the metal-ligand charge-transfer (MLCT) band of the complexes. Both complexes strongly bound to the DNA in an intercalative mode. The hypochromism (H%) of Λ-[Ru(phen)_2_(*p-*HPIP)]^2+^ and Δ-[Ru(phen)_2_(*p-*HPIP)]^2+^ were fixed at approximately 25.0% (with a 2 nm red shift) and 10.2%, respectively (**Table 1**). The spectral characteristics obviously showed that the two Ru(II) complexes interacted with DNA most likely through a mode that involves a stacking interaction between the aromatic chromophore and the DNA base pairs. In addition, the binding constant *K*_b_ and the red shift values of Λ-[Ru(phen)_2_(*p-*HPIP)]^2+^ are higher than those of Δ-[Ru(phen)_2_(*p-*HPIP)]^2+^. This result can be explained by the shallower intercalation of Δ-[Ru(phen)_2_(*p-*HPIP)]^2+^ compared with Λ-[Ru(phen)_2_(*p-*HPIP)]^2+^, which may be due to the direct hydrogen-bonding between the hydroxyl group of the *p-*HPIP ligands and the oxygen or nitrogen components of the bases as well as of the neighboring phosphate groups of DNA.

**Table 1:** Absorption spectra (λmax/nm) and hypochromism of Λ-[Ru(phen)_2_(*P-*HPIP)]^2+^ and Δ-[Ru(phen)_2_(*P-*HPIP)]^2+^.

Complexes	λmax/nm	*H*(%)	Red shift/nm	*K_b_*
Λ-Ru	458	25.0	0	8.9×10^6^ M^−1^
	283	25.9	4	
	263	30.1	2	
Δ-Ru	464	10.2	3	8.3×10^6^ M^−1^
	282	22.2	5	
	262	26.4	0	

**Figure 3:**
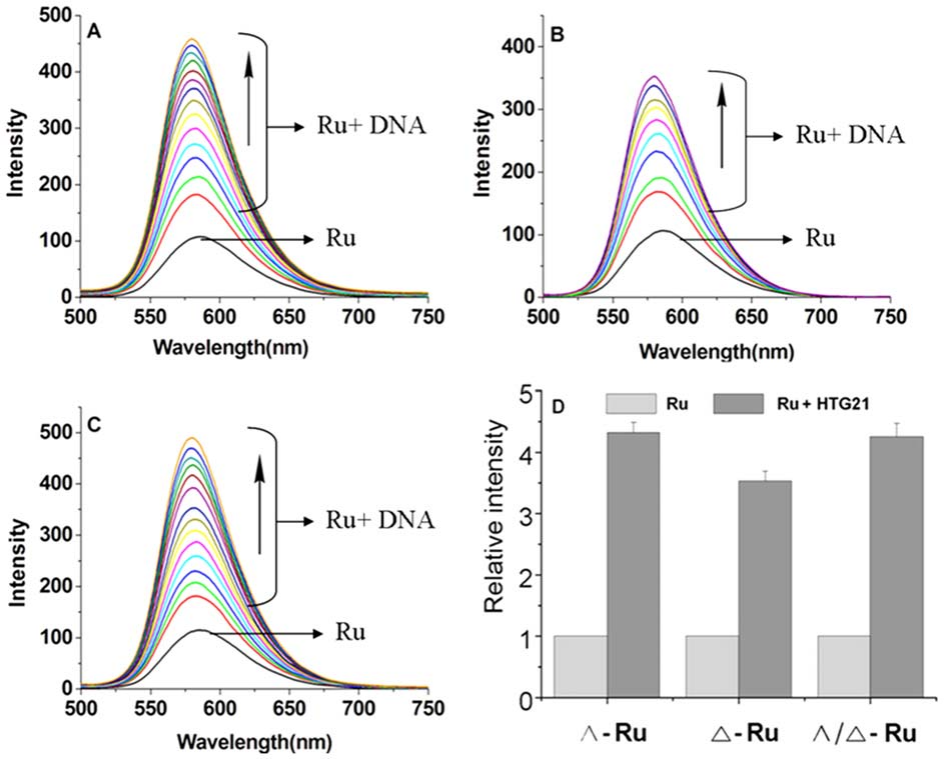
Emission spectral traces of the complexes. A)Λ-[Ru(phen)_2_(*p-*HPIP)]^2+^, b)Δ-[Ru(phen)_2_(*p-*HPIP)]^2+^, c)Λ/Δ-[Ru(phen)_2_(*p-*HPIP)]^2+^. d)Relative emission strength of Λ-[Ru(phen)_2_(*p-*HPIP)]^2+^, Δ-[Ru(phen)_2_(*p-*HPIP)]^2+^, and Λ/Δ -[Ru(phen)_2_(*p-*HPIP)]^2+^ in Tris/KCl buffer (100 mM KCl, 10 mM Tris HCl, pH 7.4) with increasing ratios of [HTG21]/[Ru] = 0∼2.5, [Ru] = 4 µM. These results are mean values of at least three independent experiments. d)Relative emission strength of Λ-[Ru(phen)_2_(*p-*HPIP)]^2+^, Δ-[Ru(phen)_2_(*p-*HPIP)]^2+^,and Λ/Δ -[Ru(phen)_2_(*p-*HPIP)]^2^.

The emission intensity of the Ru (II) polypyridyl complexes and DNA increased after their binding [38]. The emission intensities of Λ-[Ru(phen)_2_(*p-*HPIP)]^2+^, Δ-[Ru(phen)_2_(*p-*HPIP)]^2+^, and Λ/Δ-[Ru(phen)_2_(*p-*HPIP)]^2+^ increased approximately 4.32-, 3.53-, and 4.25-fold compared with the original intensities, respectively (**Figure 3d**). These results suggest that the three complexes can strongly interact with and be efficiently protected by DNA. The intrinsic binding constant *K*_b_ of Λ-[Ru(phen)_2_(*p-*HPIP)]^2+^, Δ-[Ru(phen)_2_(*p-*HPIP)]^2+^, and Λ/Δ-[Ru(phen)_2_(*p-*HPIP)]^2+^ were calculated at *K*_Λ-Ru_ = 9.3×10^5^ M^−1^, *K*_Δ-Ru_ = 7.2×10^5^ M^−1^, and *K*_Λ/Δ-Ru_ = 9.1×10^5^ M^−1^, respectively. Although the binding constant obtained from luminescence titration via the Scatchard method is different from that obtained from absorption, both sets of binding constants show that the two complexes can effectively intercalate into the DNA base pairs and that the binding ability of Λ-[Ru(phen)_2_(*p-*HPIP)]^2+^ to the quadruplex is higher than that of Δ-[Ru(phen)_2_(*p-*HPIP)]^2+^.

#### Circular dichroism spectra

**Figure 4:**
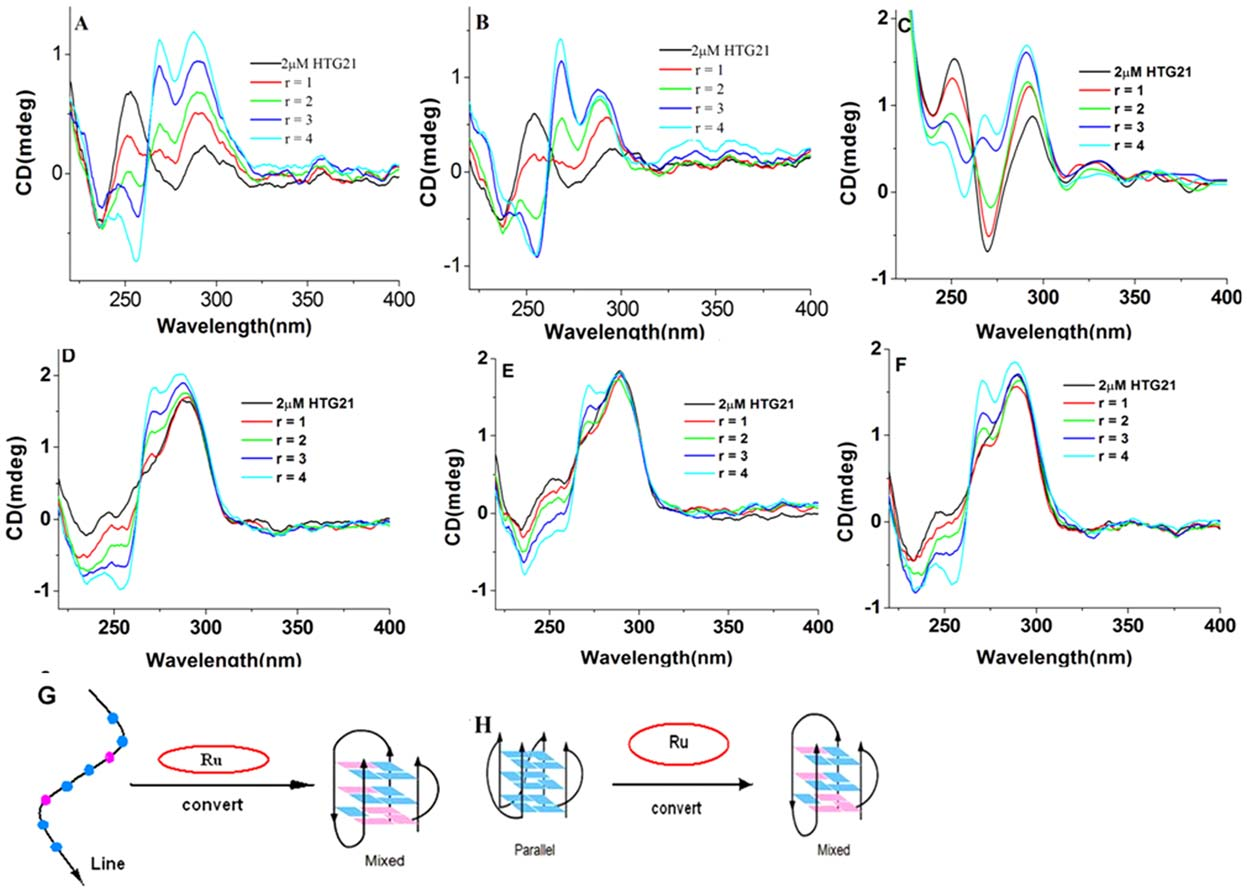
CD titration of HTG21 with complexes in 10 mM Tris buffer (pH = 7.4). a)Λ-[Ru(phen)_2_(*p-*HPIP)]^2+^, b) Δ-[Ru(phen)_2_(*p-*HPIP)]^2+^, and c) Λ/Δ-[Ru(phen)_2_(*p-*HPIP)]^2+^in 10 mM Tris buffer (pH = 7.4); d) Λ-[Ru(phen)_2_(*p-*HPIP)]^2+^, e) Δ-[Ru(phen)_2_(*p-*HPIP)]^2+^, and f) Λ/Δ-[Ru(phen)_2_(*p-*HPIP)]^2+^ in 10 mM Tris buffer, 100 mM KCl at 25°C, [HTG2] = 2 µM, [Ru] = 0∼8 µM and r : [Ru]/[HTG21]. Representative illustration of chiral ruthenium complexes induce single-strand human telomeric DNA to form a mix G-quadruplex (g) in 10 mM Tris buffer (pH = 7.4), chiral ruthenium complexes induce parallel human telomere G-quadruplex to form a mix G-quadruplex (h) in 10 mM Tris buffer, 100 mM KCl.

Circular dichroism (CD) spectroscopy was used to investigate the conformational properties of the enantiomeric chiral molecules in relation to the telomeric G-quadruplex. In the absence of salt, the CD spectrum of HTG21 at room temperature exhibited a negative band at 238 nm as well as a major positive band at 257 nm, which probably corresponds to the signal of the HTG21 random coil (characterized by a positive peak at 257 nm). A minor negative band at 280 nm and a positive band near 295 nm were also observed (**Figures 4a–4c** **, black line**) [39]. A significant change in the CD spectrum was observed upon addition of Λ-[Ru(phen)_2_(*p-*HPIP)]^2+^ to the aqueous HTG21 solution (**Figure 4a**). The bands at 257 nm gradually disappeared with the addition of the complex, eventually leading to the appearance of a major negative band at 260 nm as well as a significant increase in the band intensity at 295 nm. Meanwhile, a new, strong, positive band gradually appeared near 270 nm. These two changes are consistent with the induction of the G-rich DNA by Λ-[Ru(phen)_2_(*p-*HPIP)]^2+^ to form the G-quadruplex structure. Thus, all the complexes can convert G-quadruplex from a linear to a hybrid structure.

The HTG21 oligonucleotide formed the parallel G-quadruplex structure in the presence of K^+^ (**Figures 4d–4f** **, black line**) [40]. The CD spectrum of this structure in the absence of any compound shows a strong positive band at 290 nm, a small positive band at 260 nm, and a minor negative band at 234 nm. The CD spectrum changed upon Λ-[Ru(phen)_2_(*p-*HPIP)]^2+^ titration to the above solution, showing an enhancement of the maximum band at 290 nm as well as a suppression of the band at 260 nm. A strong, positive, induced CD signal also appeared at 270 nm. The band at 260 nm was gradually suppressed and formed a negative band until the ratio of Λ-[Ru(phen)_2_(*p-*HPIP)]^2+^ to HTG21 reached 4∶1 (**Figure 4d**). This result indicates the formation of a mixture of anti-parallel and parallel conformations, possibly including hybrid-type forms, as well. This interpretation is further supported by the recent observation of a co-existing equilibrated mixture of antiparallel, hybrid, and parallel topologies of telomeric repeats in native conditions [41]. The results also indicate that Λ-[Ru(phen)_2_(*p-*HPIP)]^2+^ is more efficient at inducing the formation of G-quadruplexes compared with the other two complexes. The data also suggest that the three complexes, particularly Λ-[Ru(phen)_2_(*p-*HPIP)]^2+^, strongly and selectively interacts with G-quadruplex DNA, which is consistent with the experimental results.

We also investigated the interactions in a Na^+^ buffer solution (**Figure S2**). The HTG21 oligonucleotide formed the antiparallel G-quadruplex structure in the presence of Na^+^. However, the CD spectrum remained nearly unchanged upon the addition of the complexes to HTG21 in the Na^+^ buffer solution. These results show that none of the three complexes changed the conformation of the antiparallel G-quadruplex in the Na^+^ solution. Therefore, Na^+^ can stabilize the conformation of the G-quadruplex, and that none of the three Ru complexes can change the conformation of the G-quadruplex at high ionic strengths [42].

The Λ-[Ru(phen)_2_(*p-*HPIP)]^2+^ and Δ-[Ru(phen)_2_(*p-*HPIP)]^2+^ complexes induced identical G-quadruplex conformation conversions in the Na^+^ and K^+^ buffer solutions. Nevertheless, we had reported that only the complex Λ-[Ru(phen)_2_(*p-MOPIP*)]^2+^ could convert the G-quadruplex conformation. Thus, the chiral isomer exhibited enantioselective binding to DNA. This result may be due to the effect of hydrogen bond as Λ-[Ru(phen)_2_(*p-*HPIP)]^2+^contains a ligand with a pendant OH functional group. The results also indicate that the interaction between different chiral Ru complexes and DNA were different.

#### Gel mobility shift assay

**Figure 5:**
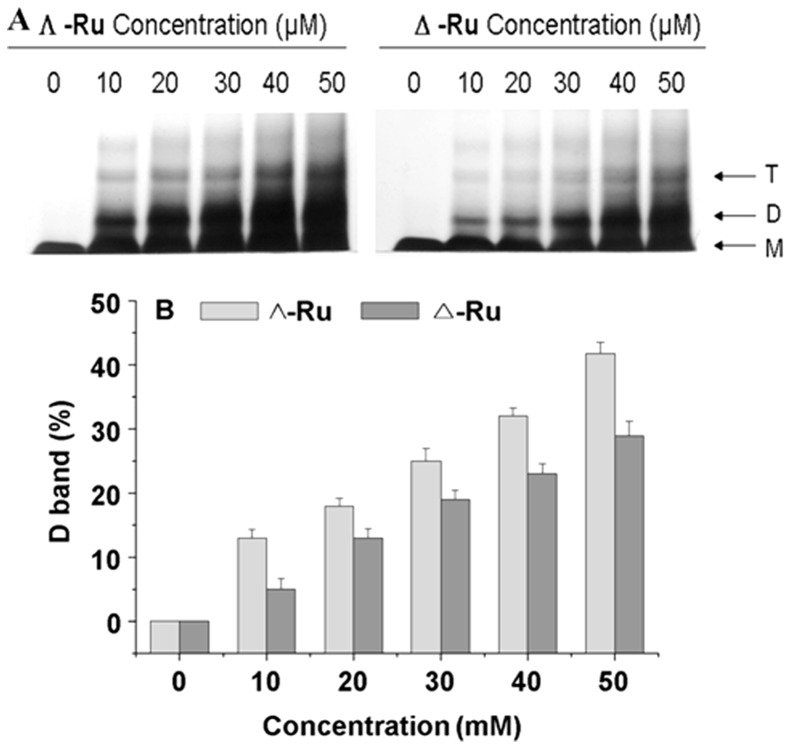
Effect of complex on the assembly of the HTG21 structure illustrated by native PAGE analysis. Ruthenium complexes at the indicated concentration were incubated with HTG21 (10 µM) at 20°C in a buffer containing 10 mM Tris, 1 mM EDTA,100 mM KCl, pH 8.0. Major bands were identified as monomer (M), dimer (D) and tetrameric (T) (a).

The ability of the Ru complexes to promote intermolecular G-quadruplex DNA formation was investigated via electrophoresis. The oligonucleotide HTG21 (5′-G_3_(T_2_AG_3_)_3_-3′) contains four repeats of the human telomeric sequence and thus has the potential to form parallel and antiparallel G-quadruplex structures in dimeric (D) and tetrameric (T) forms [43], [44]. When the HTG21 oligonucleotide was incubated in Tris buffer (10 mM Tris, 1 mM EDTA, 100 mM KCl, pH = 8.0), gel mobility shift assays show no G-quadruplex structure formation; only the band that correspond to the monomer (M) was observed. The addition of increasing amounts of Λ-[Ru(phen)_2_(*P-*HPIP)]^2+^ or Δ-[Ru(phen)_2_(*p-*HPIP)]^2+^ (from 10 µM to 50 µM) to the HTG21 oligonucleotide led to the progressive appearance of two new bands of slower mobilities; these bands correspond to the D and T G-quadruplex structures. The quantification of the gels is shown in the lower part of **Figure 5a**. The Λ-[Ru(phen)_2_(*p-*HPIP)]^2+^ complex efficiently promoted the formation of an intermolecular quadruplex structure. Up to 40% of the HTG21 oligonucleotide adopted a dimeric structure upon the addition of 50 µM Λ-[Ru(phen)_2_(*p-*HPIP)]^2+^ (**Figure 5b**). However, the treatment of the HTG21 oligonucleotide with Δ-[Ru(phen)_2_(*p-*HPIP)]^2+^ resulted in only 29% dimeric formation. These results indicate that the induction of intermolecular G-quadruplex structure formation by Δ-[Ru(phen)_2_(*p-*HPIP)]^2+^ is clearly less efficient than that of Λ-[Ru(phen)_2_(*p-*HPIP)]^2+^. These observations are consistent with the G-quadruplex stabilizing effects shown using other methods.

#### Studies of telomeric G-quadruplex binding stability and selectivity via fluorescence resonance energy-transfer (FRET) assays

**Figure 6:**
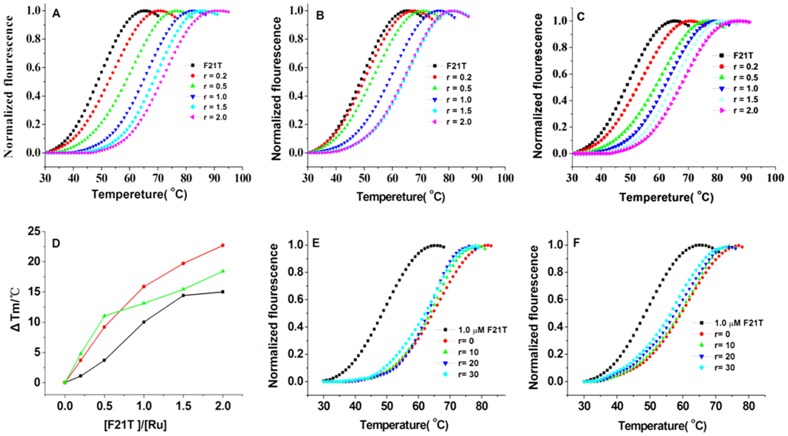
FRET melting curves for experiments carried out with F21T with Λ-Ru(a), Δ-Ru(b) and Λ/Δ-Ru(c). F21T concentration was 1 µM, in 10 mM Tris-HCl 60 mM KCl, pH = 7.4. r = [Ru]/[F21T]. (**d**): Plot of DNA stabilization temperature versus the concentration of Λ-Ru red), Δ-Ru black) and dl-Ru green) binding to F21T. Competition FRET experiment of complexes for the G-quadruplex DNA sequence over duplex DNA. Melting behavior of a G-rich oligonucleotide F21T (1 µM) alone(▪), the four other curves were obtained in the presence of complexes Λ-[Ru(phen)_2_(*p-*HPIP)]^2+^ (**e**) and Δ-[Ru(phen)_2_(*p-*HPIP)]^2+^ (**f**) (1 µM) with competitor, r = [ds26]/[F21T].

The thermodynamic stabilization activity and selectivity of the complexes to telomeric G-quadruplex DNA were investigated using FRET melting experiments [45]. We used the FRET melting assay to investigate the binding abilities of Λ-[Ru(phen)_2_(*p-*HPIP)]^2+^ and Δ-[Ru(phen)_2_(*p-*HPIP)]^2+^ to the G-quadruplex DNA F21T (FAM-G3[T2AG3]3-TAMRA, which mimics the human telomeric repeat) in 100 µM KCl buffer [46]. **Figures 6a–6c** show that in the absence of any Ru(II) complex, the DNA melting temperature (*T*_m_) of F21T in Tris/KCl buffer was 48°C. Δ*T*_m_ also gradually increased with the increased [Ru] : [DNA] concentration ratio. **Table S1** shows the *ΔT_m_* values at the concentration ratio [Ru]:[DNA = 2∶1. All three compounds significantly increased the melting temperature, indicating that these compounds have good stabilization potentials (Δ*T*_m (Λ-Ru)_ = 22.7°C, Δ*T*_m (Δ-Ru)_ = 15.0°C, and Δ*T*_m (Λ/Δ-Ru)_ = 18.4°C) for the quadruplex. The effect of the Λ-[Ru(phen)_2_(*p-*HPIP)]^2+^ complex on the G-quadruplex stability was more significant compared with those of the two other complexes. This result is consistent with those of the absorption titration studies, thereby demonstrating that Λ-[Ru(phen)_2_(*p-*HPIP)]^2+^ has the highest *Ka* value [3.87×10^5^ M^−1^] among the complexes studied. The mechanism for this behavior remains to be determined. However, the ligand of the Ru(II) complex may be vital to the stabilization.

The FRET melting experiments also provide a convenient way of testing the ligand selectivity toward the quadruplex in comparison to the selectivities toward a variety of unlabeled competitors. To determine the selectivity of the two chiral complexes, ds26 was added to quadruplex/ligand mixture as the main competitor during the experiment, given that a duplex is not labeled in the experiment. Although ds26 competes for binding to the ligand, it does not interfere in the emission studies [47]. A major advantage of this technique is that only small amounts of oligonucleotides are used, and that the experiments can be automated using a multiwell plate reader. We used the complex and F21T concentrations of 1.0 and 0.4 µM in the experiment, as well as the concentration ratios [ds26] : [F21T] = 0∶1, 10∶1, 20∶1, and 30∶1. **Figures 6e and 6f** show high levels of G-quadruplex stabilization by the chiral complexes; however, the stability was only slightly affected at the 30∶1 concentration ratio (**Figure S3**). The data also show that the chiral complexes still stabilized the G-quadruplex effectively even with the addition of substantial amounts of ds26. This result may be due to the large planar scaffold of the complexes and is consistent with the emission selectivity results, which demonstrate the high selectivity of the chiral complexes for G-quadruplex DNA over duplex DNA.

#### Polymerase chain reaction (PCR)-stop

**Figure 7:**
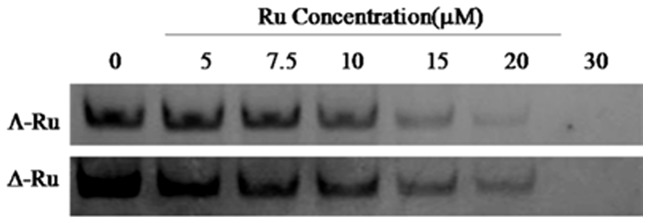
Effect of complexes on the hybridization of HTG21 in the PCR-stop assay. Λ-[Ru(phen)_2_(*p-*HPIP)]^2+^ and Δ-[Ru(phen)_2_(*p-*HPIP)]^2+^ at 0–30 µM, on the hybridization of HTG21 in the PCR-stop assay.

We evaluated the efficiency of Λ-[Ru(phen)_2_(*p-*HPIP)]^2+^ and Δ-[Ru(phen)_2_(*p-*HPIP)]^2+^ in stabilizing G-quadruplex DNA. A PCR-stop assay was used to determine whether these complexes were bound to a test oligomer [5′-G3(T2AG3)3-3′] and therefore stabilized the G-quadruplex structure [48]. In the presence of chiral complexes, the single strand HTG21 was induced into a G-quadruplex structure that blocked hybridization with a complementary strand. A 5′–3′ extension with Taq polymerase was inhibited, and the final double-stranded DNA PCR product was not detected. Different concentrations of the complexes were used in this assay. Λ-[Ru(phen)_2_(*p-*HPIP)]^2+^ showed a clearly inhibitory effect as the concentration increased from 0.0 µM to 30.0 µM, with no PCR product detected even at 20.0 µM. However, Δ-[Ru(phen)_2_(*p-*HPIP)]^2+^ showed a weaker inhibitory effect on the hybridization, eventually inhibiting the hybridization at 20 µM (**Figure 7**). These results indicate that Λ-[Ru(phen)_2_(*p-*HPIP)]^2+^ induced the stability of the G-quadruplexes better than Δ-[Ru(phen)_2_(*p-*HPIP)]^2+^. The results also indicate that G-quadruplex stabilization is vital to the inhibition of gene expression, and that all the studied complexes are efficient G-quadruplex binders.

#### Telomeric repeat amplification protocol (TRAP) assay

**Figure 8:**
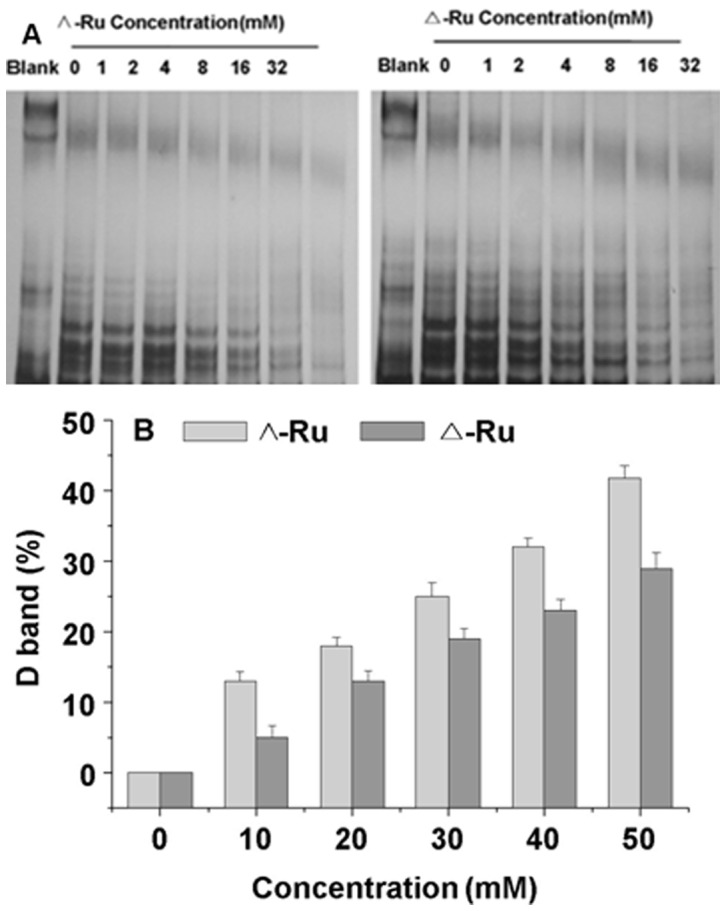
The influence of complex on the telomerase activity. Complexes Λ-[Ru(phen)_2_(*p-*HPIP)]^2+^ and Δ-[Ru(phen)_2_(*p-*HPIP)]^2+^ effected on the telomerase activity of HepG2.

The above results encouraged further investigation on the possible inhibitory effects of the two chiral Ru complexes on telomerase activity via a TRAP assay, which has been widely used to provide quantitative estimates of telomerase inhibition [49]. In this experiment, solutions containing different concentrations of Λ-[Ru(phen)_2_(*p-*HPIP)]^2+^ and Δ-[Ru(phen)_2_(*p-*HPIP)]^2+^ were added to a telomerase reaction mixture that contains HepG2 cell extracts, which express high levels of telomerase. The IC_50_ values were obtained and are shown in **vitro cytotoxicity**. **Figure 8** clearly shows the inhibitory effects of the two chiral Ru complexes on telomerase activity, but at different extents. As the Λ-[Ru(phen)_2_(*p-*HPIP)]^2+^ concentration increased, the intensity of telomerase activity decreased, particularly at 8 µM (**Figure 8**), the activity disappeared completely at 32 µM. Meanwhile, the Δ-[Ru(phen)_2_(*p-*HPIP)]^2+^ complex demonstrated inhibition at 16 µM, but this inhibition was not complete even at 32 µM. Thus, Λ-[Ru(phen)_2_(*p-*HPIP)]^2+^ has a stronger telomerase inhibitory capability compared with Δ-[Ru(phen)_2_(*p-*HPIP)]^2+^, which is consistent with the experimental data from the spectroscopic and PCR-stop analyses.

#### In vitro cytotoxicity

**Figure 9:**
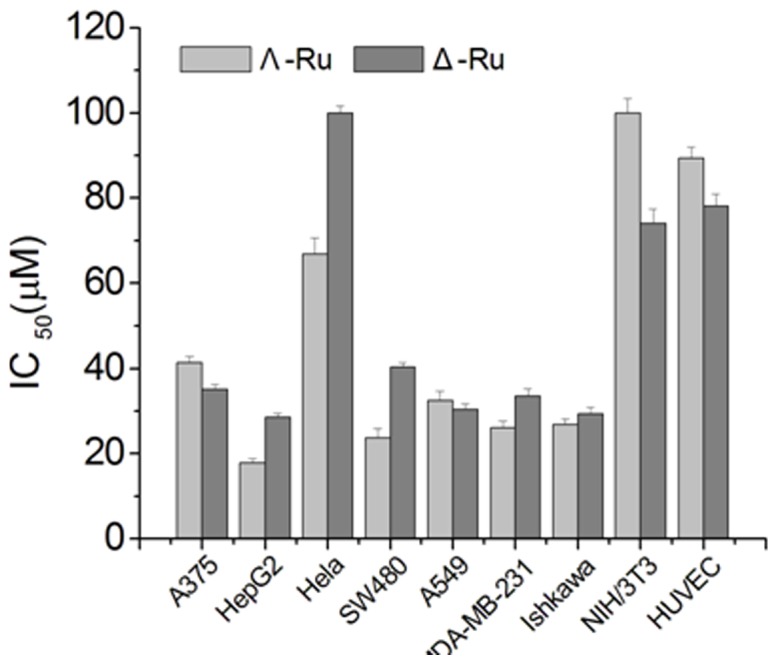
Cytotoxic effects of complexes on cells. Λ-[Ru(phen)_2_(*p-*HPIP)]^2+^ and Δ-[Ru(phen)_2_(*p-*HPIP)]^2+^ on A375, HepG2, Hela, SW480, A549, MDA-MB-231, ishkawa, and NIH/3T3 cells.

We investigated the antitumor potential of the Ru complexes using the 3–(4,5-dimethylthiazol-2-yl)-2,5-diphenyltetrazolium bromide (MTT) assay to determine the cytotoxicity of the chiral Ru(II) complexes against seven types of cancer cells, namely, human hepatocellular liver carcinoma (HepG2), human cervical cancer (HeLa), human lung carcinoma (A549), human colon colorectal adenocarcinoma (SW480), human melanoma (A375), ishkawa (endometrial adenocarcinoma), human breast cancer(MDA-MB-231) cells and human umbilical vein endothelial cells(HUVEC). All the cells were purchased from Shanghai Sangon Biological Engineering Technology & Services (Shanghai, China). **Figure 9** shows the IC_50_ values of two chiral Ru complexes after 48 h treatment. Most of the seven tested cancer cell lines were susceptible to the chiral Ru complexes, particularly the HepG2 cell. The cytotoxic activities of Λ-[Ru(phen)_2_(*p-*HPIP)]^2+^ were generally stronger than those of Δ-[Ru(phen)_2_(*p-*HPIP)]^2+^; these results are consistent with the previously described findings. The IC_50_ values of Λ-[Ru(phen)_2_(*p-*HPIP)]^2+^ toward cancer cells ranged from 17.76 µM to 66.79 µM (**Table 2**), which are significantly lower than those of Δ-[Ru(phen)_2_(*p-*HPIP)]^2+^ (28.51 µM to more than 100 µM) under the same experimental conditions and are indicative of high cytotoxicity. In particular, the two chiral complexes showed weak cytotoxicities against the human umbilical vein endothelial cells(HUVEC) with IC_50_ values at 89.35 µM and 78.12 µM. These results indicate that the complexes have relatively higher selectivity to cancer cells than to normal cells.

**Table 2:** Cytotoxic Effects of Ru Complexes towards different cell lines.(IC_50_/µM).

Complexes	IC_50_ [µM]								
	A375	HepG2	Hela	SW480	A549	MDAMB-231	Ishkawa	NIH/3T3	HUVEC
Λ-Ru	41.46±1.12	17.76±0.89	66.79±1.65	23.75±1.05	32.38±1.31	26.11±1.68	26.90±1.56	>100±3.42	89.35±2.61
Δ-Ru	35.19±1.32	28.51±1.14	>100±3.82	40.34±2.16	30.37±2.29	33.53±1.45	29.30±1.18	74.82±3.34	78.12±2.79

The anticancer activities of the two chiral Ru polypyridyl complexes in vitro demonstrate efficient enantioselection. In addition, the abilities of Λ-[Ru(phen)_2_(*p-*HPIP)]^2+^ to stabilize quadruplex DNA and inhibit telomerase were stronger than those of Δ-[Ru(phen)_2_(*p-*HPIP)]^2+^. These results suggest that the complexes may have anticancer activities, and that the quadruplex DNA and its telomerase may be the anticancer targets.

#### Cellular uptake

**Figure 10:**
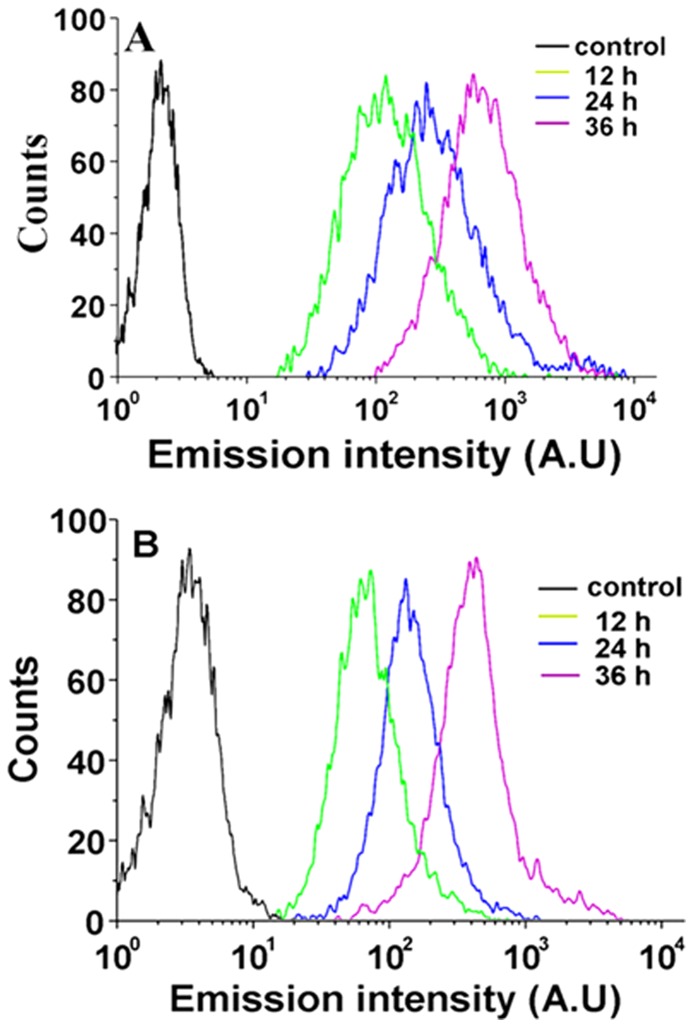
cellular uptake results of HepG2 cells. cellular uptake results of HepG2 cells incubated with blank medium (black), and complexes Λ-[Ru(phen)_2_(*p-*HPIP)]^2+^ a) and Δ-[Ru(phen)_2_(*p-*HPIP)]^2+^ b) at 37°C for 12 h (green), 24 h (blue) and 36 h (purple).

Further investigations of the complexes were conducted based on the previously described results. HepG2 cells loaded with 20 µM complexes were investigated via flow cytometry to obtain the time-dependent uptake profiles [50]. The results are shown in **Figure 10**. Upon excitation, the luminescence intensity of the cell population dramatically increased compared with the autofluorescence of untreated HepG2 cells. This result indicates the efficient cellular accumulation of the complexes. The luminescence intensity of HepG2 cells treated with Λ-[Ru(phen)_2_(*p-*HPIP)]^2+^ is stronger than that of cells treated with Δ-[Ru(phen)_2_(*p-*HPIP)]^2+^, which suggest that Λ-[Ru(phen)_2_(*p-*HPIP)]^2+^ is more effectively interiorized by the cells.

#### Confocal Microscopy Studies

**Figure 11:**
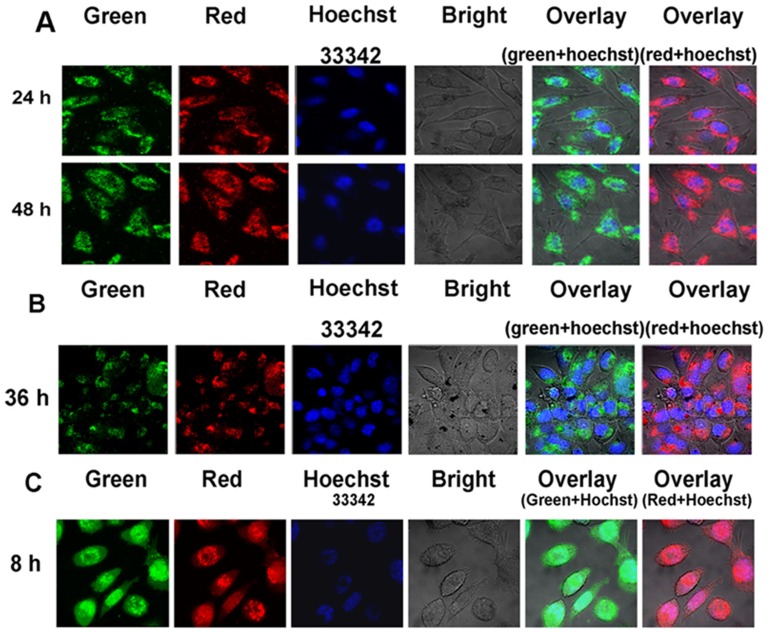
The emission imaging of the complexes entry transportation in living HepG2 cell. Emission micrographs of HepG2 cells were obtained at 24 h and 36 h after the addition of Δ-[Ru(phen)_2_(*p-*HPIP)]^2+^ (a) and Λ-[Ru(phen)_2_(*p-*HPIP)]^2+^ (b). (c) Emission imaging of Λ-[Ru(phen)_2_(p-DMNP)]^2+^ treated HepG2 cells taken by confocal microscope. (red emission from ruthenium complex, excited at 488 nm and emitted at 625–754 nm; green emission also from ruthenium complex, excited at 488 nm and emitted at 560–615 nm; blue emission from Hoechst 33342 excited at 405 nm and emitted at 420–480 nm.). Scale bar: 10 µm.

The intrinsic emission of Ru(II) complexes can be used in the design of Ru(II) complex cell-imaging probes that detect the presence of DNA binding via multiple emission peaks [20], [51]. Although some Ru(II) complexes can identify cancer cell membrane receptors and can readily accumulate in the cytoplasm of live cells,most are excluded from the nucleus and are mainly localized in the cytoplasm [52], [53]. However, a certain amount of Ru(II) complexes can be efficiently transported across the plasma membrane and then accumulate in the nucleus [54], [55]. Nuclear accumulation is highly desirable in anticancer agents that target genomic DNA [56]. The intracellular behaviors of Λ-[Ru(phen)_2_(*p-*HPIP)]^2+^ and Δ-[Ru(phen)_2_(*p-*HPIP)]^2+^ are observable via confocal microscopy. The confocal microscopic images (**Figure 11a**) show that the 20 µM Λ-[Ru(phen)_2_(*p-*HPIP)]^2+^ that were used to incubate the cells for 24 h entered and accumulated inside the cells in the region around the nucleus, subsequently forming very sharp luminescent rings around the nucleus. The nuclear region then exhibited significantly weaker emission, which is indicative of negligible nuclear uptake of the complex. Interestingly, after incubation at 20 µM for 36 h, the green/red signal in the nucleolar region increased. The complex then spread throughout the cell and partly accumulated in the nucleus. These results show that Λ-[Ru(phen)_2_(*p-*HPIP)]^2+^ can be absorbed by HepG2 cells and can enter the cytoplasm to partly accumulate in the nucleus. However, for Δ-[Ru(phen)_2_(*p-*HPIP)]^2+^, the increase in the number of green or red emission dots in the nucleus was limited (**Figure 11b**). Δ-[Ru(phen)_2_(*p-*HPIP)]^2+^ accumulated in the cytoplasm and was predominantly excluded from the nucleus after cell incubation at 20 µM for 36 h.

A similar confocal microscopic analysis was also performed using another hydrophilic Ru(II) complex, Λ-[Ru(phen)_2_(*p-*DMNP)]^2+^, which contains dimethylamino groups at the same positions on the phenyl ring as Λ-[Ru(phen)_2_(*p-*HPIP)]^2+^. After incubation of the HepG2 cells with 20 µM Λ-[Ru(phen)_2_ *(p-*DMNP)]^2+^ for 8 h, green/red emission dots were observed in the cell nuclei (**Figure 11c**). In addition, Λ-[Ru(phen)_2_(*p-*MOPIP)]^2+^ completely accumulated in the nuclei after 8 h incubation. This finding suggests that Ru complexes can enter the nucleus and efficiently interact with DNA, which leads to the inhibition of DNA transcription and translation. Therefore, the Ru compounds display promising anticancer activities. The limited capacity of Δ-Ru in nuclear targeting as well as the selective entry of Λ-Ru into HepG2 cells is also indicated by the results. The abilities of the complexes to enter the nuclei may be related to their affinities for the constituents of the nucleus as well as to differences in their photophysical properties. Furthermore, the complex containing the appropriate hydrophobic ligand may have the greater ability to enter the cells and accumulate in the nuclei.

## Conclusions

One enantiomer of a new chiral Ru(II) complex was synthesized and characterized. This enantiomer showed effective and selective binding to telomeric G-quadruplex DNA and thus inhibited the telomerase activity. The experimental results clearly show that these complexes possess certain binding affinities and significant selectivity for G-quadruplex DNA over duplex DNA. The UV/Vis, emission spectroscopy, CD spectroscopy, FRET assay, PCR-stop assay, GMSA assay, and competition experiment results all demonstrate that Λ-[Ru(phen)_2_(*p-*HPIP)]^2+^ can selectively stabilize human telomeric G-quadruplex DNA and that it has a strong preference for G-quadruplex over duplex DNA. Although the actual models for the binding of the complexes to the G-quadruplexes were not identified, our findings imply that the characteristics of the complexes that stabilize the G-quadruplexes can be further rationalized. The TRAP assay results suggest that Λ-[Ru(phen)_2_(*p-*HPIP)]^2+^ is a potential lead compound for the development of new telomerase inhibitors. These results emphasize the importance of discovering and designing chiral anticancer agents that target G-quadruplex DNA. However, Λ-[Ru(phen)_2_(*p-*MOPIP)]^2+^ was observed to have more strong ability to interact with quadruplex DNA as it contains a ligand with a methoxy group functional group, which may be involved in H-bonding interaction with the guanine in the external tetrad of G-quadruplex DNA, even the hydroxyl/methoxy group may be changed the electron density of the ligand aromatic ring atom and then the ability of complexes to interact with quadruplex DNA was different. Furthermore, the details of the binding modes of these complexes with G-quadruplex and the structure of G-quadruplex are not clear yet and further studies are needed. The activity of complexes could be adjusted by altering the functional group on the aromatic ring of the ligands.

In particular, cellular uptake and confocal microscopic results show that Λ-[Ru(phen)_2_(*p-*HPIP)]^2+^ can facilitate membrane diffusion into live cells after 24 h and partly reach the cell nucleus at 36 h. However, for Δ-[Ru(phen)_2_(*p-*HPIP)]^2+^, only diffusion into the cytoplasm was observed even after 36 h. This difference in cellular localization can be ascribed to the difference in the uptake mechanism of the two chiral complexes. The results also suggest that Λ-[Ru(phen)_2_(*p-*HPIP)]^2+^ has higher potential as a cellular nucleus-targeting drug. Moreover, although similar to the Λ-enantiomer, the hydrophobic Ru complex Λ-[Ru(phen)_2_(*p*-DMNP)]^2+^ can rapidly enter the HepG2 cell nuclei. These studies imply that the accumulation of chiral Ru complexes in the nucleus is associated with the chirality of the isomers as well as with the subtle environment of the complexes (e.g., active ligand and lipophilicity). Therefore, the nucleus is the potential cellular target of chiral Ru complexes for anticancer therapy.

## Supporting Information

Figure S1 **Absorption spectra of Λ-[Ru(phen)_2_(** ***P-*** **HPIP)]^2+^ (a) and Δ-[Ru(phen)_2_(** ***P-*** **HPIP)]^2+^ (b).** In 10 mM Tris-HCl,100 mM NaCl buffer at 25°C in the presence of increasing amounts of G-quadruplex. [Ru] = 10 µM, [DNA] = 0∼0.4 µM from top to bottom. Arrows indicate the change in absorbance upon increasing the DNA concentration.
(TIF)

Figure S2 **CD titration of HTG21 with: a)Λ-[Ru(phen)_2_(** ***P-*** **HPIP)]^2+^, b) Δ-[Ru(phen)_2_(** ***P-*** **HPIP)]^2+^, and c) Λ/Δ-[Ru(phen)_2_(** ***P-*** **HPIP)]^2+^.** In 10 mM Tris buffer, 100 mM NaCl (Ph = 7.4) at 25°C, [HTG21] = 2 µM,[Ru] = 0∼8 µM and r = [Ru]/[HTG21].d) Illustration of how chiral ruthenium complexes enantioselectively induce parallel human telomere G-quadruplex to form a mixed G-quadruplex.
(TIF)

Figure S3 **Competition FRET experiment of complexes for the G-quadruplex DNA sequence over duplex DNA.** Relative Δ*T*_m_ of Λ-[Ru(phen)_2_(*p-*HPIP)]^2+^ and Δ-[Ru(phen)_2_(*p-*HPIP)]^2+^. r = [ds26]/[F21T].
(TIF)

Figure S4 **^1^H NMR spectra of complexes** ***dl*** **- Ru(phen)_2_(** ***p*** **-HPIP)]^2+^.**
(TIF)

Figure S5 **ESI-MS and absorption spectra of complexes Λ-[Ru(phen)_2_(** ***p-*** **HPIP)]^2+^.**
(TIF)

Figure S6 **CD spectra of Δ-OH and Λ- OH in MeOH, [Ru] = 50 µM.**
(TIF)

Figure S7 **ESI-MS and absorption spectra of complexes Δ-[Ru(phen)_2_(** ***p-*** **HPIP)]^2+^.**
(TIF)

Table S1 **FRET melting curves for experiments carried out with F21T.** *ΔT*_m_ values of Λ-Ru, Δ- Ru and Λ/Δ- Ru at ratio of [Ru]/[G4] = 2, [G4] = 1 µM.
(DOC)
