# Supplementary figures and images for: Chiral Ruthenium(II) Polypyridyl Complexes: Stabilization of G-Quadruplex DNA, Inhibition of Telomerase Activity and Cellular Uptake
Source: PLoS One. 2012 Dec 7;7(12):e50902. doi: 10.1371/journal.pone.0050902 (PMC3517606; doi:10.1371/journal.pone.0050902)

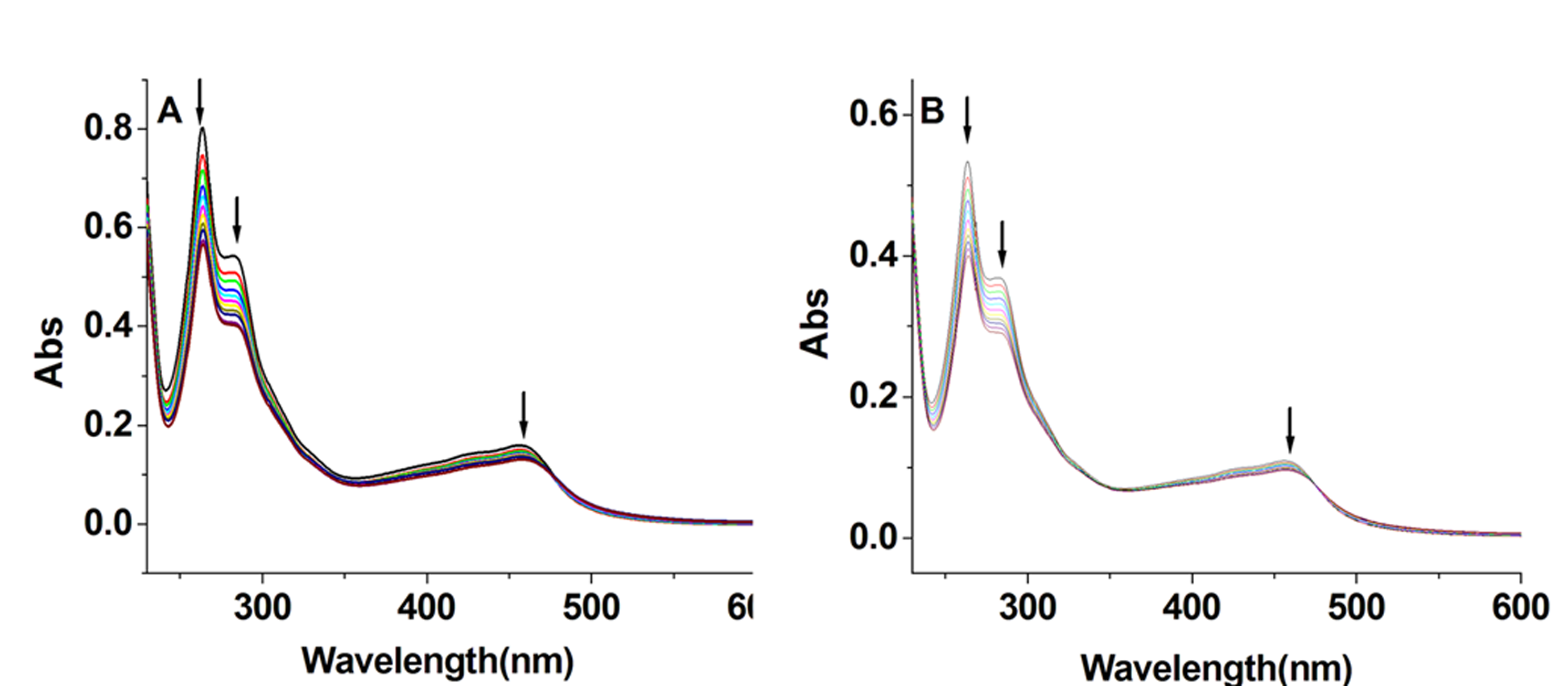

Supplement: Figure S1 — Absorption spectra of Λ-[Ru(phen)2( P- HPIP)]2+ (a) and Δ-[Ru(phen)2( P- HPIP)]2+ (b). In 10 mM Tris-HCl,100 mM NaCl buffer at 25°C in the presence of increasing amounts of G-quadruplex. [Ru] = 10 µM, [DNA] = 0∼0.4 µM from top to bottom. Arrows indicate the change in absorbance upon increasing the DNA concentration. (TIF) [file pone.0050902.s001.tif]

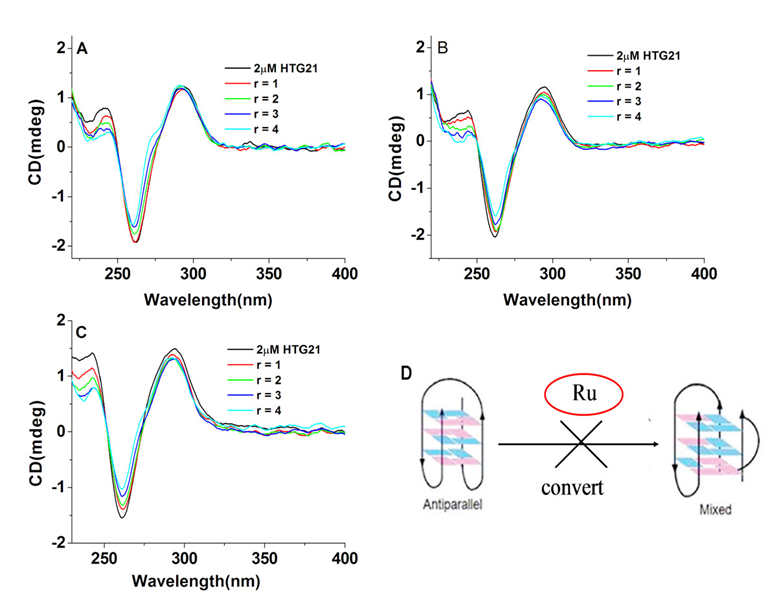

Supplement: Figure S2 — CD titration of HTG21 with: a)Λ-[Ru(phen)2( P- HPIP)]2+, b) Δ-[Ru(phen)2( P- HPIP)]2+, and c) Λ/Δ-[Ru(phen)2( P- HPIP)]2+. In 10 mM Tris buffer, 100 mM NaCl (Ph = 7.4) at 25°C, [HTG21] = 2 µM,[Ru] = 0∼8 µM and r = [Ru]/[HTG21].d) Illustration of how chiral ruthenium complexes enantioselectively induce parallel human telomere G-quadruplex to form a mixed G-quadruplex. (TIF) [file pone.0050902.s002.tif]

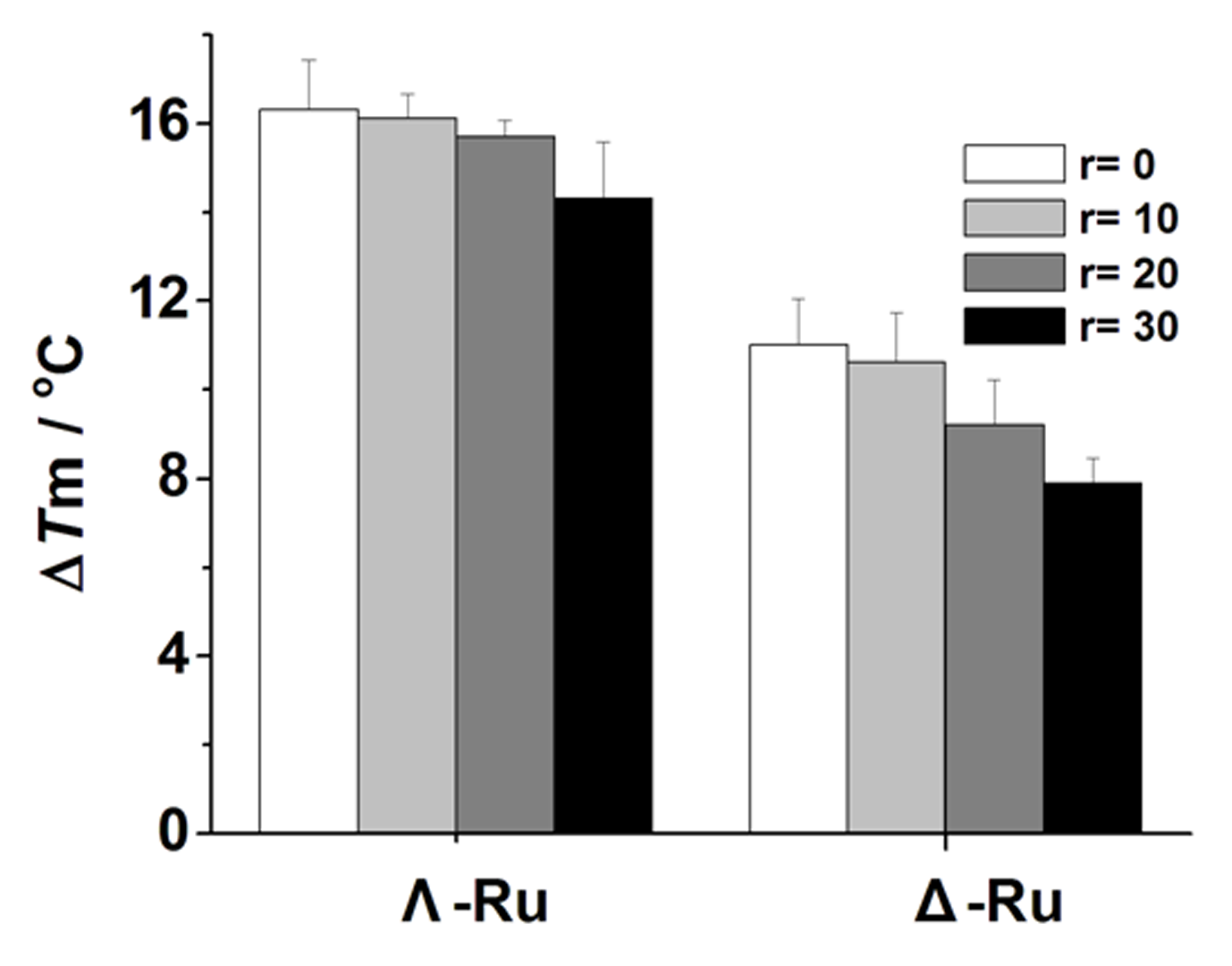

Supplement: Figure S3 — Competition FRET experiment of complexes for the G-quadruplex DNA sequence over duplex DNA. Relative ΔT m of Λ-[Ru(phen)2(p-HPIP)]2+ and Δ-[Ru(phen)2(p-HPIP)]2+. r = [ds26]/[F21T]. (TIF) [file pone.0050902.s003.tif]

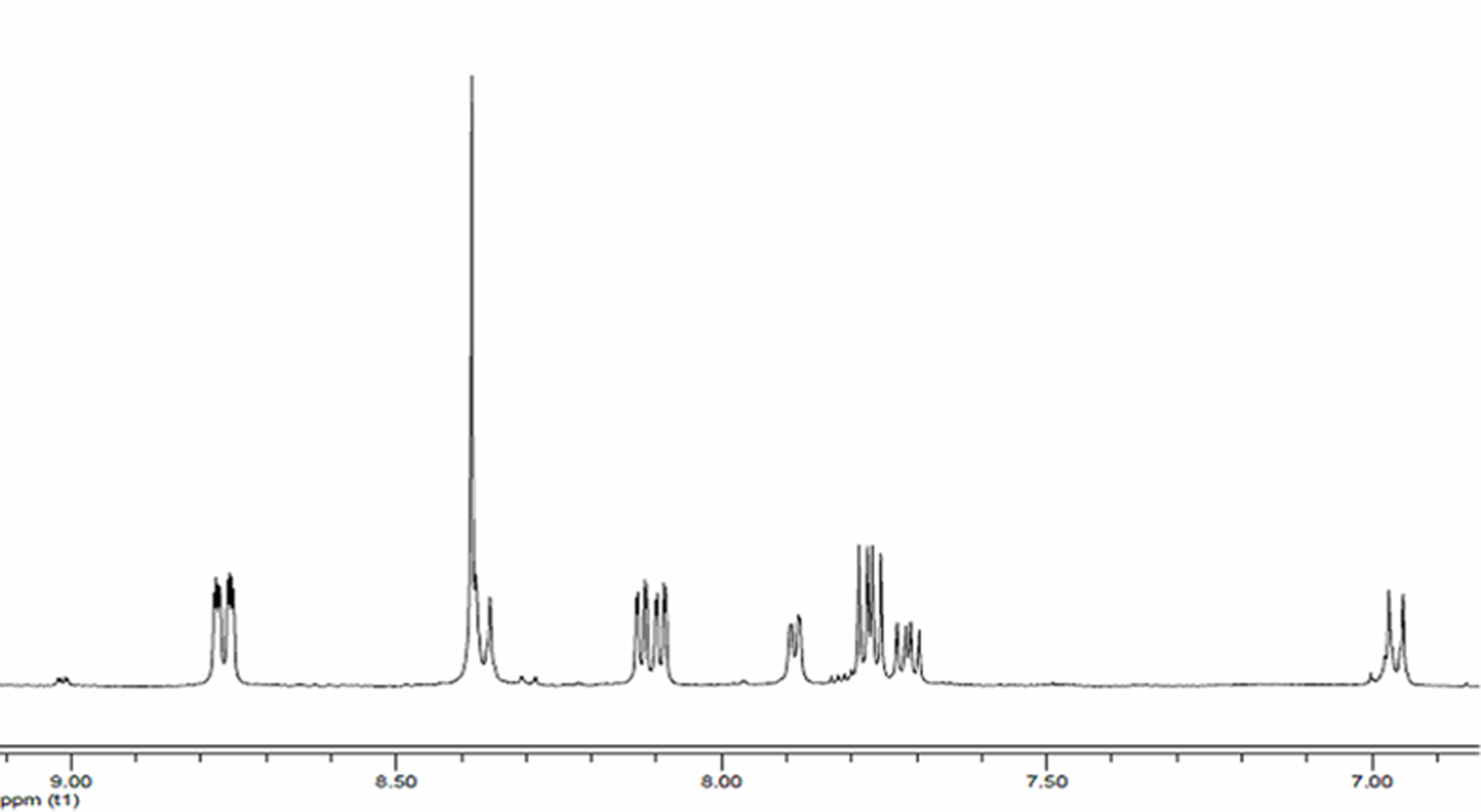

Supplement: Figure S4 — 1H NMR spectra of complexes dl - Ru(phen)2( p -HPIP)]2+. (TIF) [file pone.0050902.s004.tif]

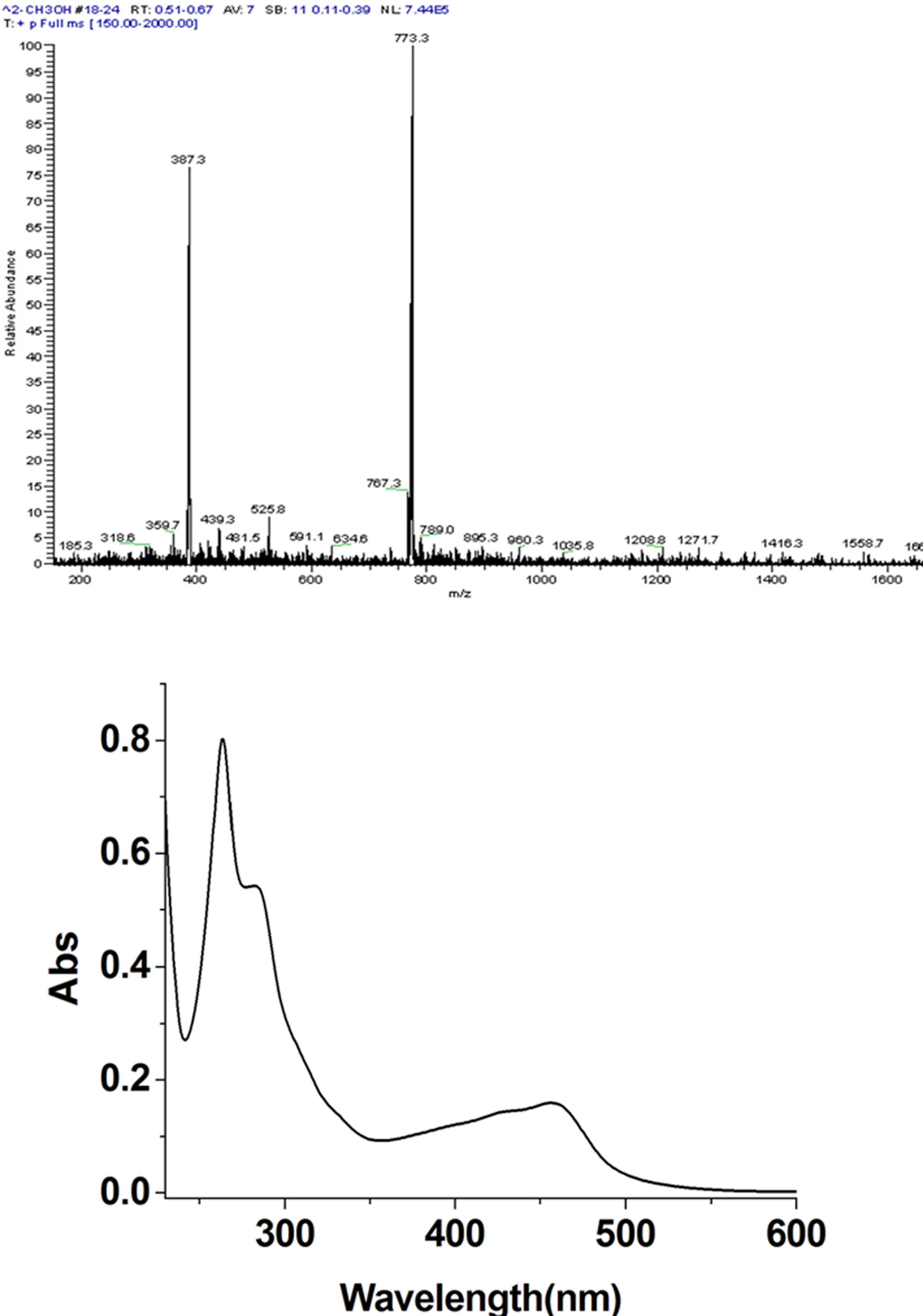

Supplement: Figure S5 — ESI-MS and absorption spectra of complexes Λ-[Ru(phen)2( p- HPIP)]2+. (TIF) [file pone.0050902.s005.tif]

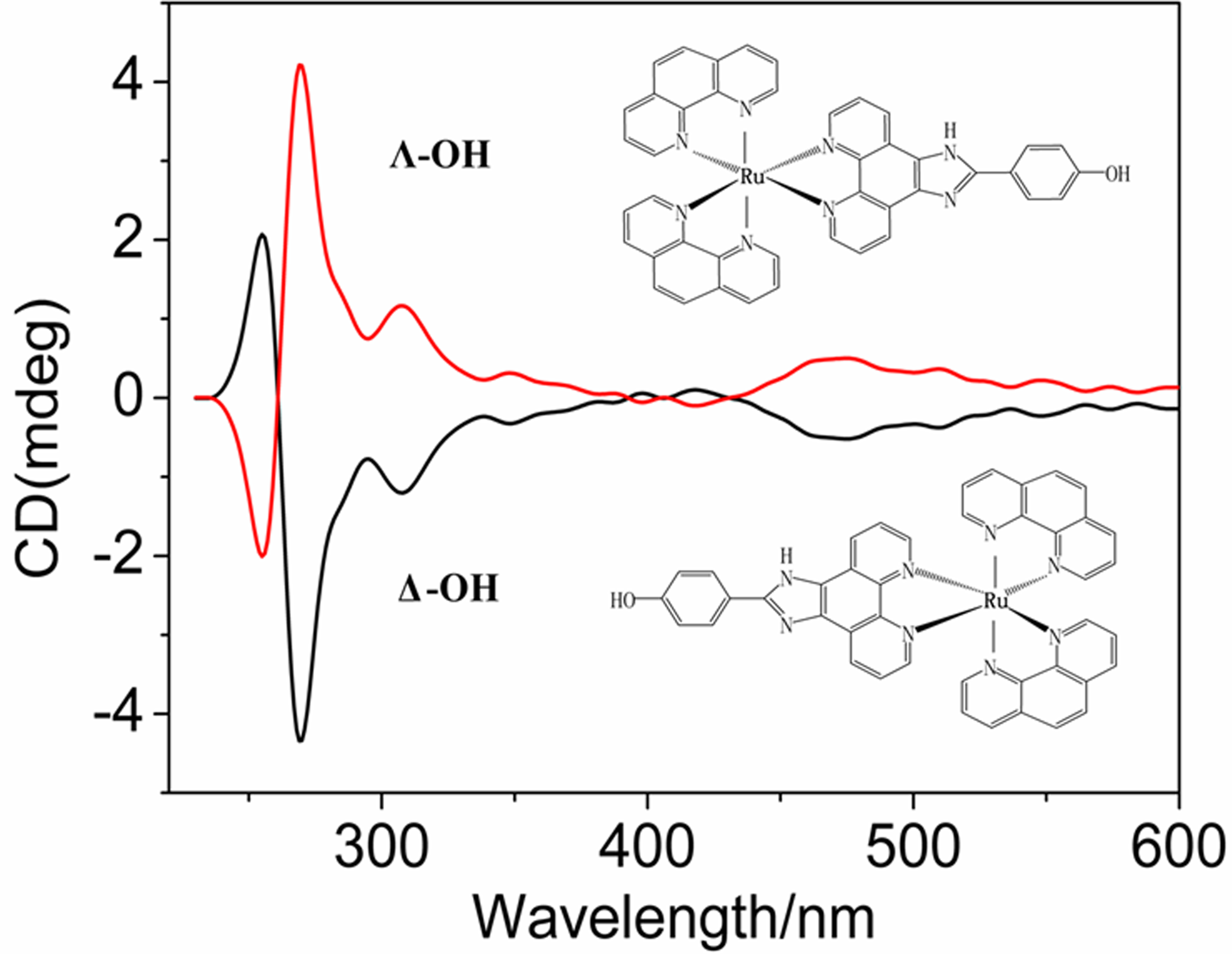

Supplement: Figure S6 — CD spectra of Δ-OH and Λ- OH in MeOH, [Ru] = 50 µM. (TIF) [file pone.0050902.s006.tif]

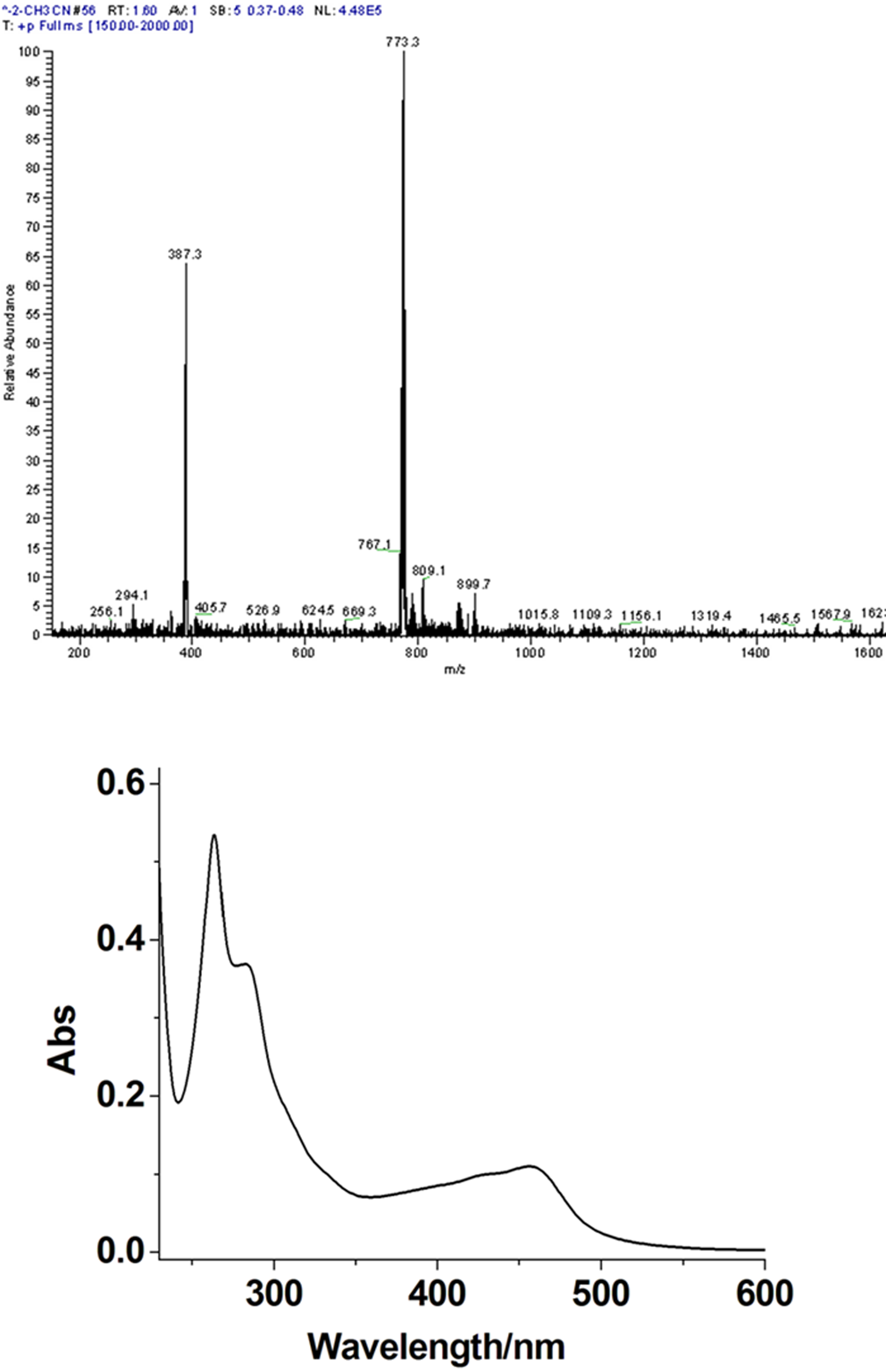

Supplement: Figure S7 — ESI-MS and absorption spectra of complexes Δ-[Ru(phen)2( p- HPIP)]2+. (TIF) [file pone.0050902.s007.tif]
