# Supplementary material for: Chiral Ruthenium(II) Polypyridyl Complexes: Stabilization of G-Quadruplex DNA, Inhibition of Telomerase Activity and Cellular Uptake
Source: PLoS One. 2012 Dec 7;7(12):e50902. doi: 10.1371/journal.pone.0050902 (PMC3517606; doi:10.1371/journal.pone.0050902)
Supplement: Table S1 — FRET melting curves for experiments carried out with F21T. ΔT m values of Λ-Ru, Δ- Ru and Λ/Δ- Ru at ratio of [Ru]/[G4] = 2, [G4] = 1 µM. (DOC) [file pone.0050902.s008.doc]

**Table S1.** FRET melting curves for experiments carried out with F21T. *ΔT*_m_ values of Λ-Ru, Δ- Ru and Λ/Δ- Ru at ratio of [Ru]/ [G4]=2, [G4]= 1 μM.

| Complexes | Λ- Ru | Δ- Ru | Λ/Δ- Ru |
| --- | --- | --- | --- |
| *ΔT*_m_(℃) | 22.7 | 15.0 | 18.4 |
